# Amorphous zinc phosphate nanoclusters loaded polycarbonate thermosensitive hydrogel: An innovative strategy for promoting wound healing

**DOI:** 10.1016/j.mtbio.2024.101266

**Published:** 2024-09-24

**Authors:** Siwen Chen, Yutong Li, Sihang Ren, Yuanyuan Yang, Zhipeng Hou, Siyu Han, Wanhong Zhang, Jing Guo, Jianshe Hu, Xing Zhang, Liqun Yang

**Affiliations:** a Center for Molecular Science and Engineering, College of Science, Northeastern University, Shenyang, 110819, PR China; b Research Center for Biomedical Materials, Engineering Research Center of Ministry of Education for Minimally Invasive Gastrointestinal Endoscopic Techniques, Shengjing Hospital of China Medical University, Shenyang, 110004, PR China; c Institute of Metal Research, Chinese Academy of Sciences, Shenyang, 110016, PR China; d Department of Plastic Surgery, The Second Hospital of Dalian Medical University, Dalian, 116027, PR China; e Liaoning Research Institute for Eugenic Birth & Fertility, China Medical University, Shenyang, 110031, PR China

**Keywords:** Thermosensitive hydrogel, Polycarbonate, Amorphous zinc phosphate, Vascularization, Wound healing

## Abstract

Skin trauma is a matter of great concern for public health, emphasizing the importance of reconstructing the microenvironment at the trauma site to facilitate tissue regeneration. Therefore, the investigation of innovative wound dressings has significant research and clinical implications. In this study, we prepared a thermosensitive hydrogel based on a hydrophilic-hydrophobic-hydrophilic triblock polycarbonate polymer (PTP), and created a composite hydrogel, PTPH-AZP, by incorporating amorphous zinc phosphate (AZP) nanoclusters. We evaluated the effects of PTPH-AZP on human umbilical vein endothelial cells (HUVECs) and the ability to promote skin wound healing. According to the results, PTPH-AZP was found to promote the proliferation, migration, and tube formation of HUVECs through the sustained release of Zn^2+^ at appropriate concentrations. *In vivo* experiments demonstrated that in the early-mid stages of wound healing, PTPH-AZP promotes increases in Platelet Endothelial Cell Adhesion Molecule-1 (CD31) and α-Smooth Muscle Actin (α-SMA) content within the wound area, facilitating accelerated re-epithelialization and enhanced collagen deposition. In later healing stages, epidermal thickness in the PTPH-AZP treated group was significantly improved, aligning with surrounding intact skin with no instances of attenuated or hypertrophic scarring observed. The findings from the *in vivo* study suggested that PTPH-AZP may have a positive impact on vascularization and wound healing. In conclusion, this study presents a promising strategy for skin wound healing, highlighting the potential of PTPH-AZP as an effective therapeutic approach.

## Graphical abstract

**Figure:**
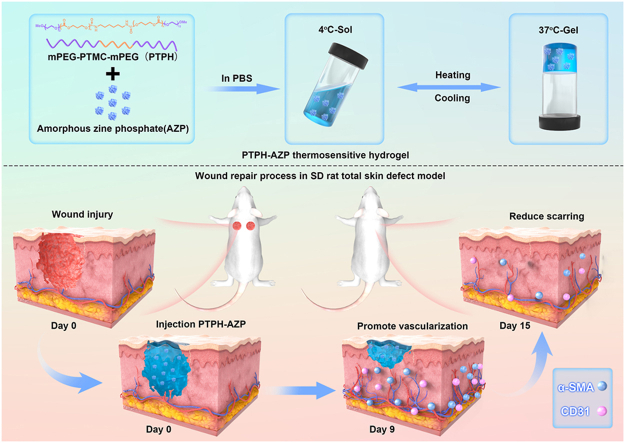

## 1 Introduction

The skin, an intricate organ comprising the epidermis, dermis, and subcutaneous tissue, serves pivotal functions like sensation, thermoregulation, and defense against external elements [1,2]. Its vulnerability to damage underscores the importance of wound healing, a multi-stage process encompassing coagulation, inflammation, angiogenesis, cell proliferation, tissue remodeling, and scar formation [3,4]. Effective angiogenesis is crucial for tissue regeneration post-injury, emphasizing the need to stimulate vascular regrowth for oxygen and nutrient supply essential for cell repair [[5], [6], [7]].

Zinc (Zn) is a transition metal that is found in significant amounts in the human body. It plays an essential role in human growth, development, and immune response. Additionally, Zn^2+^ is a key component of several proteins and serves as a vital coenzyme in tissue repair [8,9]. During wound healing, Zn^2+^ acts as a critical cofactor in activating matrix metalloproteinases (MMPs), enzymes crucial for promoting collagen deposition and vascular remodeling [10,11]. Zinc phosphate (Zn_3_(PO_4_)_2_), an inorganic material, has garnered significant attention due to its excellent biocompatibility, biosafety, and environmental compatibility. It has found applications in diverse fields such as antimicrobial agents, antiseptics, glass-ceramics, dental cement, tissue engineering, and drug delivery systems [[12], [13], [14], [15]]. However, further research is needed to investigate its potential application in wound healing. Previous studies have indicated that an optimal concentration of Zn^2+^ can promote angiogenesis [16]. However, excessively high concentrations of Zn^2+^ can be toxic to fibroblasts and vascular endothelial cells, highlighting the importance of controlled release [17,18]. To effectively control the release concentration of Zn^2+^ and ensure uniform nanoparticle dispersion while mitigating the risks of localized toxicity, it is crucial to select an appropriate carrier and loading strategy [19].

Hydrogels, three-dimensional polymeric networks formed through physical or chemical crosslinking [[20], [21], [22]], are widely employed in biomedical applications, particularly as carriers for drugs or nanoparticles [23,24]. Their biocompatibility, high water content, and softness, make them ideal materials for wound dressings [[25], [26], [27], [28]]. However, conventional pre-formed hydrogel dressings frequently struggle to conform to irregularly shaped wounds. Injectable hydrogels address this limitation by undergoing a sol-gel transition in response to environmental stimuli, such as changes in pH, temperature, or photo-induced crosslinking, Schiff base formation, Michael addition, and other mechanisms [26,[29], [30], [31], [32], [33]]. Among these, thermosensitive hydrogels, which exhibit a sol-gel transition upon increasing temperature, stand out as particularly promising. They eliminate the need for small-molecule crosslinking agents or organic solvents, making them highly versatile. Thermosensitive hydrogels can serve as therapeutic agents, carriers for bioactive molecules or cells, and efficient drug delivery systems or three-dimensional cell growth scaffolds [34,35].

Polyethylene glycol (PEG), a hydrophilic polymer renowned for its excellent biocompatibility, is widely employed in the design and synthesis of thermosensitive injectable hydrogels. The development of biodegradable thermosensitive hydrogels based on PEG has been a significant area of research, often achieved through the incorporation of hydrophobic chain segments such as polycaprolactone (PCL), polylactic acid (PLA), poly(lactic-co-glycolic acid) (PLGA), and other aliphatic polyesters [36]. These hydrogels find numerous applications in the biomedical field, particularly for drug, DNA, and stem cell delivery [[37], [38], [39], [40], [41], [42]]. Aliphatic polycarbonates also recognized for their excellent biocompatibility and biodegradability, offer distinct advantages over polyesters in biomedical applications due to their ability to degrade without producing acidic byproducts [43,44]. Kim et al. previously synthesized mPEG-PTMC diblock copolymers by copolymerizing mPEG with TMC in a specific ratio, resulting in a thermo-responsive hydrogel sensitive to human body temperature [45]. While previous research has predominantly focused on the applications of the amphiphilic polymer mPEG-PTMC in drug delivery, its potential for wound healing applications has received limited attention [[46], [47], [48], [49], [50]]. PTMC-based thermosensitive hydrogels exhibit several properties that make them promising candidates for wound healing, including temperature sensitivity at human body temperature, adaptability to irregular wound geometries, and the ability to maintain wound moisture.

**Scheme 1:**
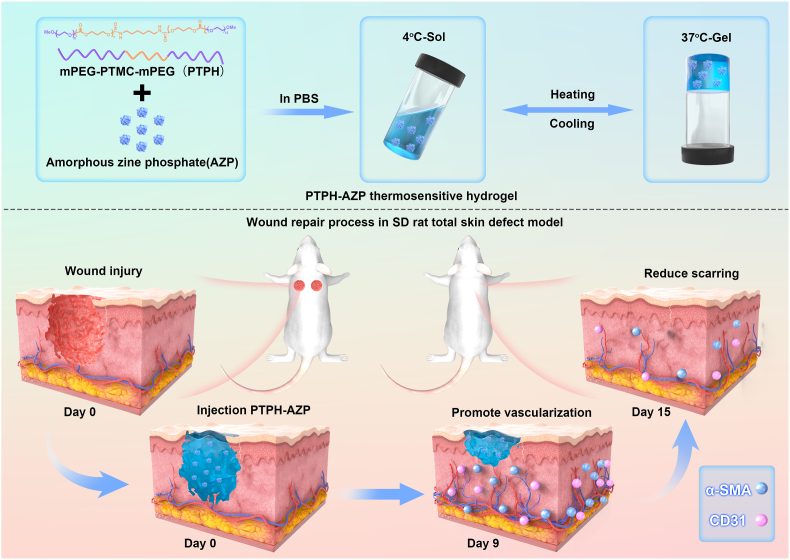
Schematic of preparation and application of PTPH-AZP.

To optimize the properties of aliphatic polycarbonate temperature-sensitive hydrogels and broaden the applications in wound healing, we developed a novel human thermosensitive hydrogel called PTPH-AZP. This hydrogel is composed of mPEG_12_-PTMC_n_-mPEG_12_ (PTP) triblock copolymer with a hydrophilic-hydrophobic-hydrophilic structure and AZP nanoclusters. In this study, we evaluated the angiogenic efficacy of PTPH-AZP by assessing the proliferation, migration, and angiogenic effects of PTPH-AZP-treated HUVECs. *In vivo* experiments were conducted on full-thickness skin defect models in SD rats to explore the efficacy of PTPH-AZP in promoting wound healing and potential mechanisms. The specific protocol of the study was detailed in Scheme 1.

## 2 Materials and methods

### 2.1 Materials

Trimethyl carbonate (TMC, purity ≥99.8 %) was sourced from Jinan Biotechnology Co., Ltd., China. Polyethylene glycol (PEG, M_n_ = 20000, purity ≥99 %), monomethoxy polyethylene glycol (mPEG_12_, M_n_ = 550, purity ≥99.5 %), hexamethylene diisocyanate (HDI, purity ≥98 %), and tin(II) 2-ethylhexanoate (purity ≥99.8 %) were supplied by Sigma Aldrich. Dichloromethane, toluene, and ether (purity ≥99.5 %) were provided by Sinopharm Chemical Reagent Co., Ltd. Ammonia water (NH_3_.H_2_O, 25.0–30.0 %), and Phosphate Buffered Saline (PBS, pH = 7.4) were obtained from Macklin. Diammonium hydrogen phosphate ((NH_4_)_2_HPO_4_, purity ≥99 %), zinc chloride (ZnCl_2_, purity ≥99 %), and polyacrylic acid (PAA, purity ≥99 %) were provided by Aladdin. Lipase solutions (from Thermomyces lanuginosus, ≥100,000 U/g) were purchased from Sigma-Aldrich. Toluene was dehydrated through azeotropic reflux with sodium wire before use. All other solvents used in this study did not require further purification.

### 2.2 Synthesis and characterization of PTPH-AZP

#### 2.2.1 Synthesis route of PTPH-AZP

AZP nanoclusters were prepared following our previous research method [51]. The synthesis steps were briefly as follows: Firstly, 4.08 g of ZnCl_2_ and 1.28 g of PAA were dissolved in 500 mL of ultrapure water, and the pH was adjusted to approximately 3.3 using NH_3_·H_2_O. Concurrently, 2.64 g of (NH_4_)_2_HPO_4_ and 0.2 g of PAA were dissolved in 500 mL of ultrapure water, with the pH adjusted to about 9.0. The above two solutions were then mixed to achieve a pH of 6.5 and stirred rapidly at room temperature for 1 h. After stirring, the system was added to a 20 % polyethylene glycol (M_w_: 20000) aqueous solution for 6 h. Subsequently, the mixture was dialyzed against ultrapure water for 36 h, with the dialysis fluid being replaced every 12 h. Finally, the dialysis solution was freeze-dried for 72 h using a lyophilizer. The resulting AZP powder was stored at −20 °C for further use.

The synthesis steps of PTP are as follows: initially, a specific ratio of dried mPEG_12_ (Initiator) and TMC (Monomer) was mixed with a toluene solution of tin (II) 2-ethylhexanoate (Catalyst) in a polymerization tube. The air in the system was then replaced with N_2_, repeating the process three times to ensure the complete removal of toluene and air. Subsequently, the polymerization tube was heat-sealed under high vacuum conditions (5 mmHg), and the system was stirred at 130 °C for 24 h to obtain mPEG_12_-*b*-PTMC_n_ (PT). After the reaction, HDI was added to the mixture and stirred at 60 °C for 6 h. To remove any unreacted monomers, the polymer was dissolved in dichloromethane and gradually added to cold ether for precipitation and purification. The final product was vacuum-dried and refrigerated, resulting in a yield of 72 %. The resulting mPEG_12_-PTMC_n_-mPEG_12_ triblock copolymers were named PTP-1, PTP-2, and PTP-3, while the unconjugated mPEG_12_-*b*-PTMC_n_ was named PT-3. Further information can be found in Table 1.

**Table 1:** Reaction ratio between PT and PTP and molecular weight.

Group	Monomer: Initiator	Monomer: Catalyst	M_n_^a^	M_n_^b^	M_W_/M_n_^b^
PTP-1	17:1	250:1	5460	5278	1.63
PTP-2	18:1	250:1	5588	5486	1.52
PTP-3	19:1	250:1	5768	5811	1.66
PT-3	19:1	250:1	2875	2977	1.44

a Determined by ^1^H NMR.

b Determined by GPC.

Prepared AZP was dispersed in PBS (pH = 7.4) and subjected to ultrasound for 30 min. Subsequently, a PBS solution containing mPEG_12_-PTMC_n_-mPEG_12_ polymer (40 %, w/w) was added at 4 °C to prepare PTPH-AZP1 (AZP, 0.5 %, w/w) and PTPH-AZP2 (AZP, 2 %, w/w).

#### 2.2.2 Characterization methods

The chemical structure was characterized by ^1^H Nuclear Magnetic Resonance (^1^H NMR, Bruker ARX 600, Germany) and Fourier Transform Infrared Spectroscopy (FT-IR, PerkinElmer Spectrum One (B) spectrometer, USA). The thermodynamic properties were characterized using Differential Scanning Calorimetry (DSC, Netzsch DSC-204, Germany) and Thermogravimetric Analysis (TGA, Netzsch 209C, Germany). The microstructure was observed using Scanning Electron Microscopy (SEM, HITACHI SU8010, Japan) and Transmission Electron Microscopy (TEM, FEI, Hillsboro, Tecnai G2 F20, U.S.A.). The molecular weight and molecular weight distribution of the polymers were obtained through Gel Permeation Chromatography (GPC, Waters Model 1515, USA). Energy Dispersive X-ray Spectroscopy (Ultim Extreme, Oxford Instruments, UK) was used in conjunction with SEM (HITACHI SU8010, Japan) to analyze the elemental distribution of the AZP nanoparticles. The hydrodynamic size and Zeta potential were measured using a Zetasizer Nano Particle Analyzer (Malvern, U.K.) employing the Dynamic Light Scattering (DLS) technique. The crystalline structure of AZP nanoparticles was analyzed using an X-ray Diffractometer (XRD, Rigaku D/max 2400 diffractometer, Tokyo, Japan).

### 2.3 Sol-gel transition of hydrogels

The sol-gel transition temperature of the hydrogel was determined using the inversion method and dynamic mechanical analysis. For the inversion method, vials containing 0.5 mL of PTPH were immersed in a water bath at 4 °C for 30 min. The PTPH system underwent a gradual temperature increase of 1 °C. After each increment, the vial was inverted to observe the transition temperature. The transition temperature was determined based on the criterion of flow (sol) to no-flow (gel). Each data point represents the average of three measurements. The dynamic mechanical analysis of PTPH was conducted using a dynamic rheometer (Anton Paar MCR 302, Austria). Parallel plates with a diameter of 20 mm and a gap of 0.5 mm were used to hold the PTPH-3 (40 %, w/w). The heating rate was set at 0.2 °C/min, and data collection was performed at a frequency of 1.0 rad/s and a controlled stress of 4.0 dyn/cm^2^. Furthermore, the viscosity of the hydrogel was studied at 37 °C to assess its dependence on the shear rate (0.01–100 rad/s). A strain sweep test was conducted on PTPH-AZP, where the strain was increased from 0.1 % to 1000 % at a constant angular frequency of 10 rad/s at 37 °C. Subsequently, strain step cycles between 1 % and 1000 % were performed at 37 °C and 10 rad/s for a total of five cycles.

### 2.4 Testing of PTPH-AZP adhesion

The adhesive strength of PTPH-AZP was qualitatively evaluated using a lap shear test on porcine skin. Fresh porcine skin was initially soaked in saline solution to remove the fat layer and then cut into rectangular blocks. The hydrogel was injected between two porcine skin samples, covering an area of 40 mm × 20 mm. After adhesion, the samples were maintained at 37 °C for 10 min to ensure complete sol-gel transition. Subsequently, the lap shear test was conducted in tensile mode using an electronic universal testing machine (WDW-02) at a tensile rate of 10 mm/min. The maximum tensile strength was recorded at the conclusion of the test.

### 2.5 *In vitro* degradation experiment of PTPH

Degradation experiment was performed by modifying a previously described protocol [52]. Details can be found in the SI.

### 2.6 *In vitro* release analysis of zinc ions from PTPH-AZP

To assess the release profile of zinc ions, 0.5 mL of PTPH-AZP1, PTPH-AZP2, and PTPH were separately placed in 5 mL centrifuge tubes. The tubes were incubated at 37 °C for 10 min to allow for hydrogel formation. Subsequently, 1.5 mL of phosphate-buffered saline (PBS, pH 7.4) was added to each tube. The samples were then incubated on a shaking incubator at 37 °C with a shaking rate of 60 rpm, with gentle shaking for 8 h per day. Supernatants were collected at 0.5, 1, 3, 6, 12, 24, 48, and 72 h, filtered through a 0.22 μm pore size filter, and digested. The concentration of zinc ions was measured using inductively coupled plasma mass spectrometry (ICP-MS, Agilent 7850, USA).

### 2.7 Cell culture, proliferation, scratching assay, and tube formation

HUVEC and mouse fibroblasts (L929 cells) proliferation was evaluated using the Cell Counting Kit-8 (CCK-8, Biosharp, China), and the migration and tube formation ability of PTPH-AZP on HUVECs was investigated. Detailed experimental procedures were described in the SI.

### 2.8 Hemolysis tests

Hemolysis testing was performed by modifying a previously described protocol [53]. Details can be found in the SI.

### 2.9 Evaluation of *in vivo* biocompatibility of PTPH-AZP and the efficacy in treating skin defects in SD rats

The *in vivo* biocompatibility of PTPH-AZP was assessed through subcutaneous implantation experiments in SD rats. Initially, 9 healthy male SD rats (weighing 220–250 g) were anesthetized following standard procedures, and the dorsal area was shaved. Subsequently, 0.5 mL of PTPH-AZP or saline was injected subcutaneously into the rats using a syringe. Epithelial tissue samples from the implantation site were collected on day 3 and 7.

A full-thickness skin defect model was chosen to comprehensively evaluate the performance of PTPH-AZP. The wound area was photographed on day 0, 3, 6, 9, 12, and 15, calculated using Image J. The exact procedure is described in SI. All animal experiments were strictly conducted by the Animal Management Regulations of the Ministry of Health of the People's Republic of China (Document No. 55, 2001) and institutional guidelines, and were approved by the Ethics Committee of the Liaoning Research Institute for Eugenic Birth and Fertility.

### 2.10 Histology and immunohistochemistry

Regenerated skin tissues were collected on day 9 and 15. They were fixed in 10 % paraformaldehyde, dehydrated through a graded series of alcohols, embedded in paraffin, and sectioned. The wound sections were stained with Hematoxylin and Eosin (H&E, Sigma-Aldrich) and Masson's trichrome (Sigma-Aldrich). The proportion of collagen deposition was evaluated by analyzing the intensity of the blue areas in Masson staining using Image J software. To assess the impact of PTP-AZP on angiogenesis, immunohistochemistry (IHC) was performed on the regenerated skin tissue samples collected on day 9 and 15 for CD31 and α-SMA detection. The images were scanned using a slide scanner (Aperio CS2). Vessel numbers and positive areas were determined by counting three randomly selected areas based on CD31 staining.

### 2.11 Quantitative real-time PCR (qRT-PCR) assay

Skin tissue samples were collected and homogenized on day 9 and 15, following the manufacturer's protocol (TRIzol; Ambion, USA) for total RNA extraction. The extracted RNA was then converted into cDNA using a cDNA synthesis kit (Kangwei Co., China). Real-time quantitative PCR was performed to evaluate the relative expression levels of the α-SMA gene, with GAPDH used as an internal reference. Each experiment was conducted in triplicate, and the data are presented as mean values. The primers used for α-SMA were as follows: Forward Primer: CATCATGCGTCTGGACTTGG, Reverse Primer: CCAGGGAAGAAGAGGAAGCA.

### 2.12 Western blotting assay

Regenerated skin tissues from the wound sites were collected on day 9 and 15 post-surgery. The tissues were washed 2–3 times with PBS to remove any remaining blood. Subsequently, the tissues were cut into small pieces and placed in a homogenizer. RIPA lysis buffer (Thermo Fisher Scientific Inc, USA) was added to the tissue under ice bath conditions to ensure thorough homogenization. The extract was then centrifuged at 12000 g for 10 min, and the protein concentration was determined using a BCA Protein Assay Kit. The target proteins were separated through electrophoresis and transferred onto a polyvinylidene fluoride (PVDF) membrane. To block the PVDF membrane, it was incubated in a mixture of 5 % BSA prepared in skim milk at room temperature. The incubation was carried out with shaking at 60–70 rpm for 1 h, followed by washing with TBST buffer. The membrane was then incubated overnight with a primary antibody against α-SMA (ab5694, Abcam, USA). After three washes with TBST, the membrane was incubated for 1 h with a goat anti-rabbit secondary antibody (ab6721; 1/2000, Abcam, USA). Following another wash with TBST, the membrane was imaged using a gel imaging system. GAPDH was used as the internal reference.

### 2.13 Statistical analysis

Data were presented as mean ± standard deviation. Statistical significance was analyzed using one-way analysis of variance (ANOVA). Significant differences were considered when ∗*p* < 0.05, ∗∗*p* < 0.01, and ∗∗∗*p* < 0.001.

## 3 Results and discussion

### 3.1 Preparation and characterization of PTP

**Fig. 1:**
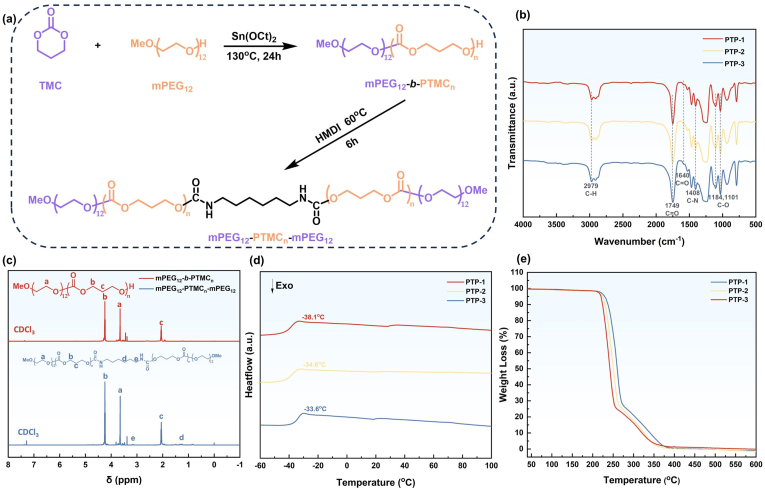
(a) Synthesis scheme of PTP. The structure of PTP was determined by (b) FT-IR spectra and (c) ^1^H NMR spectra, while the thermodynamic properties were determined by (d) DSC curves and (e) TGA curves.

The synthetic route of PTP is illustrated in Fig. 1 (a). The process involves a two-step reaction. Firstly, ring-opening polymerization of TMC is initiated by mPEG_12_, resulting in the formation of the diblock copolymer mPEG_12_-*b*-PTMC_n_. Subsequently, mPEG_12_-*b*-PTMC_n_ was coupled with HDI to produce the triblock copolymer PTP. The structure of PTP was characterized by FT-IR spectroscopy. The spectrum in Fig. 1 (b) reveals characteristic absorption peaks confirming the successful synthesis of the triblock copolymer. The peaks at 1184 and 1101 cm⁻^1^ are attributed to the C-O-C stretching vibrations in the mPEG_12_ segment, while the peak at 2979 cm⁻^1^ corresponds to the C-H stretching vibrations of CH_2_ in the polymer chain segment. The presence of the characteristic C=O stretching vibration of the polycarbonate segment at 1749 cm⁻^1^ and the amide bond at 1640 cm⁻^1^ confirms the successful polymerization of TMC initiated by the terminal hydroxyl group of mPEG_12_. Additionally, the absorption peak at 1404 cm⁻^1^ corresponds to the C-N stretching vibration of the amide bond, further validating the successful coupling of mPEG_12_-*b*-PTMC_n_ with HDI. These findings collectively confirm the successful synthesis of the triblock copolymer PTP. The structure of the synthesized triblock copolymer was further elucidated using ^1^H NMR spectroscopy. As shown in Fig. 1 (c), the signal at 3.60 ppm is attributed to -CH_2_ protons of mPEG_12_. The -CH_2_ protons of PTMC are represented by signals at 2.00 and 4.10 ppm. Peaks observed at 1.28 and 3.21 ppm are attributable to the two types of CH_2_ protons in HDI. These findings corroborate the intended molecular design of PTP. The molecular weight of PTP in each group was determined by GPC (Fig. S1), with the specific molecular weights summarized in Table 1. The difference in molecular weight between PT-3 and PTP-3 before and after HDI coupling provides further confirmation of the successful coupling reaction, leading to the formation of PTP. The thermal properties of PTP polymers were characterized by DSC and TGA. The glass transition temperature (T_g_) of a polymer is a critical parameter that determines its physical state (glassy or highly elastic) under different application conditions. As shown in Fig. 1 (d), the T_g_ valued for PTP-1, PTP-2, and PTP-3 were determined to be −38.1 °C, −34.6 °C, and −33.6 °C, respectively. The absence of a melting peak during the heating process indicates the amorphous nature of the PTP polymer. Notably, the T_g_ of PTP was below the physiological temperature of the human body (37 °C), suggesting that it will remain soft and pliable at body temperature. This characteristic makes PTP a promising candidate for hydrogel applications, where a soft and flexible material is desirable. The decomposition temperature (5 % weight loss) of PTP is an important factor to consider when selecting sterilization methods. In Fig. 1 (e), the decomposition temperatures of all three polymer groups exceed 200 °C, demonstrating excellent thermal stability of PTP. This high thermal decomposition temperature allows for the use of conventional high-temperature sterilization methods, such as autoclaving, without compromising the structural integrity of PTP. This feature is advantageous as it offers a cost-effective and widely accessible sterilization option.

### 3.2 Preparation and characterization of AZP

**Fig. 2:**
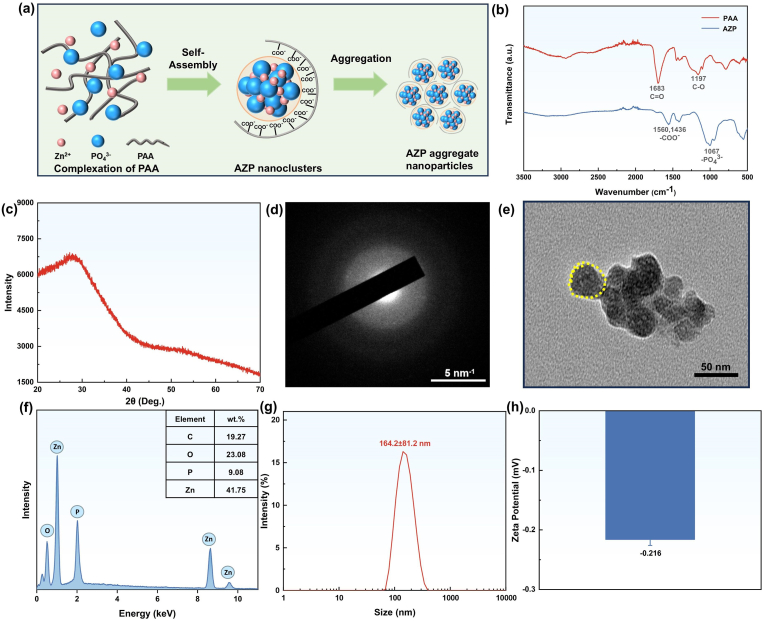
(a) Presented a schematic diagram illustrating the progressive assembly process of AZP nanoclusters. (b) FT-IR spectra of PAA and AZP. (c) XRD patterns of the synthesized AZP nanoclusters. (d) The electron diffraction pattern of AZP. (e) An image captured using the TEM bright field technique displays AZP nanoclusters. (f) SEM/EDS analysis was performed on AZP nanoclusters. (g) The particle size distribution of AZP nanoclusters. (h) The zeta potential distribution of AZP nanoclusters.

The synthesis of AZP nanoclusters is illustrated in Fig. 2 (a). A solution of Zn^2+^ dispersed in PAA was gradually added to a phosphate solution under thorough mixing. Electrostatic interactions between PO_4_^3−^ and Zn^2+^ led to the formation of initial AZP nanoclusters. As the AZP nanoclusters coexisted with a significant number of PAA molecules, they self-assembled and gradually aggregated, ultimately forming amorphous AZP nanoclusters. The FT-IR spectra of PAA and AZP in the wavenumber range of 3500–500 cm^−1^ are presented in Fig. 2 (b). The peak observed at 1683 cm^−1^ was attributed to the C=O stretching vibration, while the peak at 1197 cm^−1^ represented the C-O stretching vibration of PAA [54]. The characteristic peak of AZP at 1067 cm^−1^ is attributed to the stretching vibration of the PO_4_^3−^ group. Upon chelation of Zn^2+^ ions with PAA, the formation of the -COO- group is evident due to strong coupling between the C=O and C-O bonds, resulting in stretching vibration peaks at 1436 cm^−1^ and 1560 cm^−1^ [51]. These findings support the formation of hybrid nanoclusters through the chelation of PAA molecules with Zn^2+^ ions. The properties of the AZP nanoclusters were further examined. The XRD spectrum showed broad peaks, indicating that the synthesized AZP nanoclusters are amorphous (Fig. 2 (c)). This observation was corroborated by the electron diffraction pattern obtained from a selected area of the AZP nanoclusters using TEM, which displayed diffused rings with blurred boundaries, confirming their amorphous nature (Fig. 2 (d)). TEM images revealed both individual AZP nanoclusters and their aggregates (Fig. 2 (e)). The size of the AZP nanoclusters ranged from 25 nm to 40 nm, while the aggregated AZP nanoclusters measured approximately 150 nm in size. The results from the EDS analysis revealed that the AZP nanoclusters consist of Zn (41.75 wt%), O (23.08 wt%), C (19.27 wt%), and P (9.08 wt%) (Fig. 2 (f) and S2). These nanoclusters were dispersed in the aqueous phase and had a hydrodynamic size of 164.2 ± 81.2 nm (Fig. 2 (g)). The zeta potential of the AZP nanoclusters was measured to be −0.216 mV, indicating a negatively charged surface, which makes them prone to coagulation (Fig. 2 (h)).

### 3.3 Gel transition and rheological properties of PTPH and PTPH-AZP

**Fig. 3:**
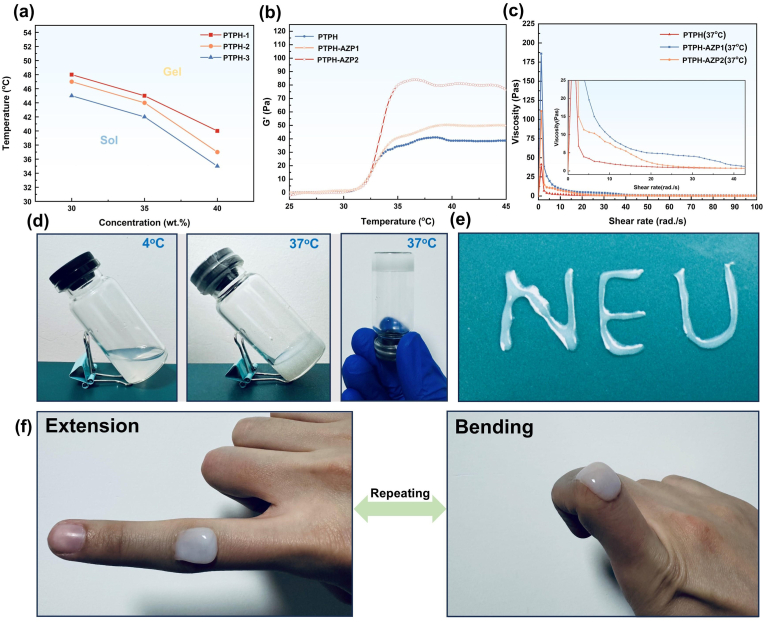
Studies of the gel transition temperature and rheological behavior of PTPH: (a) sol-gel transition temperatures of PTPH. (b) Changes in the storage modulus with temperature for PTPH, PTPH-AZP1, and PTPH-AZP2. (c) Relationship between viscosity and shear rate in the gel state for PTPH, PTPH-AZP1, and PTPH-AZP2. (d) Investigation of the gelation behavior using the tube inversion method. (e) Injectable properties of PTPH. (f) Skin adhesiveness of PTPH-AZP: finger extension and finger bending.

The sol-gel transition in the PBS solution of PTP (PTPH) was achieved by partially dehydrating of the mPEG_12_ chain segments, enhancing the hydrophobic interactions of PTMC blocks via heating [45]. Different concentrations of PTPH (30 %, 35 %, and 40 % w/w), prepared using PTP synthesized in different proportions as shown in Table 1, were analyzed to determine the sol-gel transition temperatures of each group (Fig. 3 (a)). PTPH-2 (40 % w/w) and PTPH-3 (40 % w/w) both exhibited a sol-gel transition at 37 °C. PTPH-3 (40 % w/w) displayed a slightly lower sol-gel transition temperature (∼34–35 °C), rendering it suitable for environments slightly below body temperature. This property makes PTPH-3 (40 % w/w) an ideal candidate for hydrogels designed for irregularly shaped skin wounds. Therefore, the PTPH-3 (40 % w/w) solution was selected for constructing the PTPH-AZP thermosensitive hydrogel. The rheological properties of the hydrogels were evaluated using rheometry. The modulus changes of different hydrogel groups were initially measured at a fixed frequency of 1 Hz under varying temperatures (Fig. 3 (b)). A sharp increase in the storage modulus (G′) of all hydrogel groups was observed as the temperature rose to 33–34 °C, indicating a sol-gel transition within the system. This is due to the hydrophobic association between PTMC and the hydrophobicity-enhancing segments of mPEG_12_ after the temperature rises [36,55]. Notably, PTPH-AZP, formed by incorporating AZP nanoclusters into PTPH, exhibited the same temperature-induced sol-gel transition behavior as PTPH. At 37 °C, the G′ of PTPH was approximately 39.5 Pa. Upon the addition of AZP nanoclusters, the G′ of PTPH-AZP1 increased to about 45.9 Pa. Further increasing the content of AZP nanoclusters from 0.5 % to 2 % (w/w %) resulted in a significant rise in the modulus of PTPH-AZP2 to approximately 83.8 Pa. This phenomenon is likely attributed to the enhanced overall rigidity of the PTPH system following the incorporation of AZP nanoclusters. To further understand the rheological properties, the relationship between viscosity and shear rate was investigated. As shown in Fig. 3 (c), the viscosity of the hydrogels reached a peak at approximately 1 rad/s. This behavior can be attributed to the hydrophobic interactions within the hydrogels, which resist shear stress at low shear rates. However, as the shear rate exceeded 1 rad/s, these hydrophobic interactions were disrupted, leading to a significant decrease in viscosity. This observation suggests that the hydrogels exhibit shear-thinning behavior, transitioning from a viscous gel-like state to a more fluid-like state under applied shear forces. This unique characteristic, coupled with the biocompatibility of PTPH-AZP, makes it a promising candidate for injectable and minimally invasive delivery and coverage of irregular wounds. In order to comprehensively evaluate the mechanical properties and recovery behavior of PTPH-AZP, we performed repeated dynamic strain step tests at 37 °C, utilizing strain amplitudes of 1 % and 1000 %, at a frequency of 10 rad/s (Fig. S3). The results demonstrate that a 1000 % strain significantly disrupted the hydrogel network. However, after five cycles of repeated scanning, the modulus of PTPH-AZP could be restored to its initial value under a 1 % strain. This observation suggests that PTPH-AZP exhibits remarkable self-healing properties. This self-healing behavior can be attributed to the reversible aggregation of hydrophobic segments during the sol-gel transition of the amphiphilic polymer hydrogel. The hydrophobic segments act as reversible crosslinking points, which can reassemble after the 1000 % strain scanning, enabling the self-healing property of the PTPH-AZP [53]. The sol-gel transition temperature of PTPH-AZP was determined using the tube inversion method, as illustrated in Fig. 3 (d), to evaluate its gelation behavior. The results indicated that PTPH-AZP undergoes a sol-gel transition at 37 °C. Furthermore, Fig. 3 (e) demonstrated the successful formation of the ‘NEU’ pattern through syringe injection at 37 °C, confirming the excellent injectability of PTPH-AZP. Additionally, the adhesion of PTPH-AZP to the skin was evidenced by the absence of fracturing or detachment when applied to finger joints and subjected to repetitive flexion and extension (Fig. 3 (f)). The stress-strain curve from the lap shear test of PTPH-AZP adhered to porcine skin at 37 °C demonstrated a maximum shear stress of 6.89 ± 1.62 kPa. This indicates that PTPH exhibits excellent skin adhesive properties. This adhesion capability is likely attributed to the increased viscosity of the hydrogel at 37 °C, as well as hydrogen bonding interactions between ether bonds and ester carbonyl groups in the polymer chains and amino, hydroxyl, and carboxyl groups on the skin surface (Fig. S4). The excellent injectability and skin adhesion properties of PTPH-AZP highlight its promising application prospects in wound healing The microstructure of PTPH-AZP was investigated using SEM, revealing its characteristic hydrogel porous features (Fig. S5). Typically, hydrogels exhibit a well-defined three-dimensional porous architecture. However, due to the low molecular weight, amorphous, and non-crystalline nature of PTP polymers, along with the inherent creep properties, most of the pores cannot be sustained over time and eventually collapse due to creep. Consequently, the complete three-dimensional porous structure of PTPH-AZP is not fully captured in the SEM images. As a degradable aliphatic polycarbonate hydrogel, investigation of its degradation properties is crucial. Given that macrophages at the wound site may produce lipase, potentially influencing the degradation rate of aliphatic polycarbonates, we subjected PTPH-AZP to both hydrolytic (PBS, pH = 7.4) and enzymatic degradation with 0.1 % (w/w) lipase in PBS (Fig. S6) [56]. The presence of lipase accelerated the degradation rate of PTPH compared to degradation in PBS alone, although both groups exhibited similar degradation trends within 7 days. Notably, PTPH-AZP degradation remained stable during the first three days, fulfilling the clinical requirement of a 3-day dressing change cycle for sustained Zn^2+^ release and enhanced wound healing.

### 3.4 *In vitro* cell proliferation and migration assay of PTPH-AZP

**Fig. 4:**
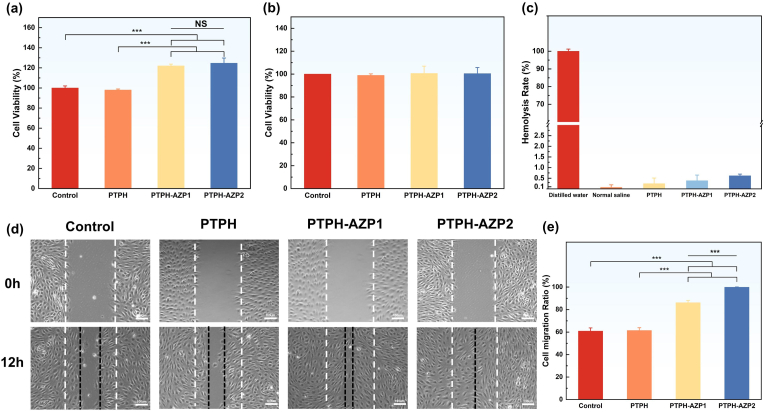
Assessment of the proliferative capabilities of hydrogel cell culture medium impregnation solutions on (a) HUVECs and (b) L929 cells. (c) Hemolysis experiments of the hydrogels. (d, e) Impact of hydrogel cell culture medium impregnation solutions on cell migration. Each value is presented as mean ± SD (n = 3). ∗*p* < 0.05, ∗∗*p* < 0.01, ∗∗∗*p* < 0.001.

The *in vitro* release of Zn^2^⁺ from PTPH-AZP1 and PTPH-AZP2 was investigated in phosphate-buffered saline (PBS, pH = 7.4) (Fig. S7). An initial burst release of Zn^2^⁺ was observed within the 3 h, with concentrations of approximately 7.86 ± 1.38 μM and 15.49 ± 0.97 μM released from PTPH-AZP1 and PTPH-AZP2, respectively. This initial release can be attributed to the release of surface-enriched AZP nanoclusters from the PTPH. Subsequently, the release of Zn^2^⁺ tended to stabilize at 24 h, indicating the controlled release effect of the PTPH on the AZP nanoclusters. The average Zn^2^⁺ release concentrations from PTPH-AZP1 and PTPH-AZP2 were approximately 3.52 ± 0.89 μM and 8.72 ± 1.07 μM, respectively. Notably, the overall Zn^2^⁺ release concentrations are known to promote HUVECs proliferation, suggesting potential for further investigation of PTPH-AZP1 and PTPH-AZP2 in wound healing applications [17]. To evaluate the *in vitro* biocompatibility of AZP, PTPH, and PTPH-AZP, we conducted cell viability assays using the Cell Counting Kit-8 (CCK-8). Under conditions simulating Zn^2^⁺ release in PBS, each material was incubated in the culture medium for 24 h, followed by co-culturing with HUVECs and L929 cells for 72 h. The impregnation solutions were initially co-cultured with HUVECs. Fig. 4(a) showed that the cell viability in the PTPH group was approximately 98.1 %, indicating no proliferative effect on HUVECs. A viability above 95 % signifies good biocompatibility, demonstrating no notable toxicity to HUVECs. Conversely, PTPH-AZP1 and PTPH-AZP2 showed significantly enhanced HUVEC proliferation, with cell viabilities of approximately 122.1 % and 124.7 %, respectively, compared to the control group. This substantial proliferative effect suggests a potential role for PTPH-AZP in promoting angiogenesis during skin wound healing. Subsequently, the toxicity and proliferation of each group of cell culture medium impregnation on L929 cells were investigated (Fig. 4 (b)). Cell viability assays revealed no significant differences among the groups, suggesting that neither PTPH nor PTPH-AZP exhibited apparent cytotoxicity towards L929 cells nor stimulated their proliferation. This finding, in conjunction with the observed promotion of HUVEC proliferation, highlights the favorable biocompatibility of PTPH-AZP and its potential for wound healing applications. Notably, the ability to promote angiogenesis while minimizing excessive fibroblast proliferation suggests that PTPH-AZP could facilitate tissue regeneration and potentially reduce scar formation. It is worth mentioning that the results indicated that AZP exhibited good biocompatibility with L929 cells at concentrations below 50 μg/mL in the culture medium, while concentrations exceeding 100 μg/mL result in significant cytotoxicity to L929 cells (Fig. S8). There is a significant difference between the biosafe concentration of AZP (100 μg/mL) and the required concentration of PTPH-AZP1 (5 mg/mL), attributable to the sustained release effect of PTPH on AZP. Due to the substantial disparity in concentrations, subsequent experiments did not use AZP alone as a control. Instead, the focus was on evaluating the proliferative and migratory capabilities of PTPH-AZP on HUVECs. Hemolysis tests is a standard assessment of biomaterial compatibility with red blood cells, were conducted to evaluate the potential for hemolytic reactions upon contact with PTPH and PTPH-AZP. The results consistently demonstrated hemolysis rates below 5 %, indicating a negligible impact on red blood cells and confirming the excellent biocompatibility of both hydrogels (Fig. 4 (c)). To assess the influence of PTPH and PTPH-AZP on endothelial cell migration, a cell scratch assay was performed using HUVECs cultured in a cell culture medium impregnated with the respective materials (Fig. 4(d and e)). Compared to the control group (60.90 ± 2.80 %) and the PTPH group (61.45 ± 2.44 %), both PTPH-AZP1 (86.10 ± 1.95 %) and PTPH-AZP2 (99.83 ± 0.29 %) significantly enhanced HUVEC migration, indicating a potential for promoting angiogenesis. Notably, the PTPH-AZP2 group exhibited the most pronounced cell migration behavior, with the cell scratch almost completely healed. These results underscore the potential of PTPH-AZP, particularly PTPH-AZP2, to promote angiogenesis, a critical process in wound healing and tissue regeneration.

### 3.5 Endothelial tubulogenesis *in vitro*

**Fig. 5:**
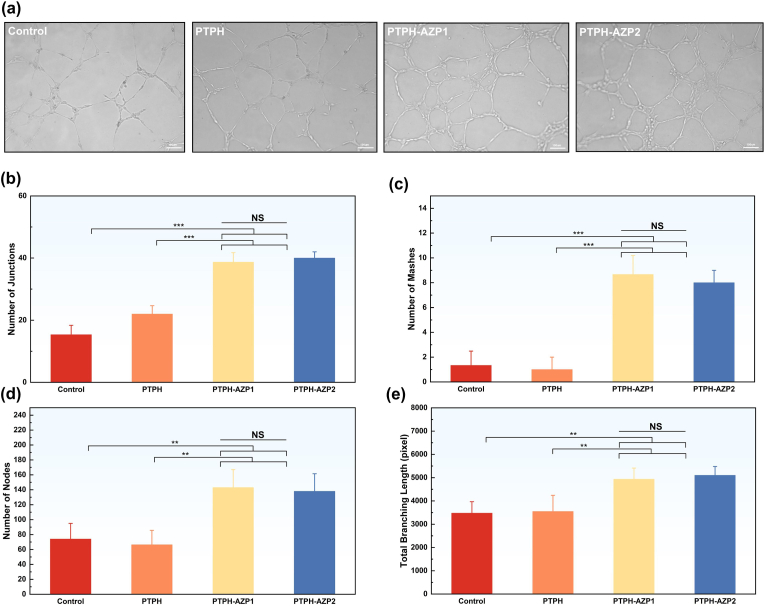
(a) Presents optical images of tube formation after co-culturing HUVECs with PTPH, PTPH-AZP1, and PTPH-AZP2 cell culture medium impregnation solutions. Quantification was conducted by calculating (b) the junctions, (c) meshes, (d) nodes, and (e) the total branching length. Each value is presented as mean ± SD (n = 3). ∗*p* < 0.05, ∗∗*p* < 0.01, ∗∗∗*p* < 0.001.

Microvascularization is a critical aspect of regenerative medicine and tissue engineering, facilitating tissue regeneration and repair [57]. To explore the potential of PTPH-AZP in promoting angiogenesis, we investigated its effects on HUVEC proliferation, migration, and further conducted an *in vitro* tube formation assay. HUVECs were co-cultured with PTPH, PTPH-AZP1, and PTPH-AZP2-impregnated cell culture medium on a matrix gel surface for 6 h (Fig. 5(a–e)). While the PTPH group showed no significant difference in angiogenic capacity compared to the control group, both PTPH-AZP1 and PTPH-AZP2 groups exhibited a significant enhancement in vascular formation, characterized by increased capillary density and branching structures. Notably, the PTPH-AZP2 group demonstrated the most prominent formation of interconnected capillary-like structures with higher levels of junctions, meshes, nodes, and total branching length compared to the control group. These findings highlight the exceptional potential of PTPH-AZP, particularly PTPH-AZP2, to promote angiogenesis, a crucial process in wound healing and tissue regeneration.

### 3.6 Evaluation of the efficacy of PTPH-AZP on wound healing

**Fig. 6:**
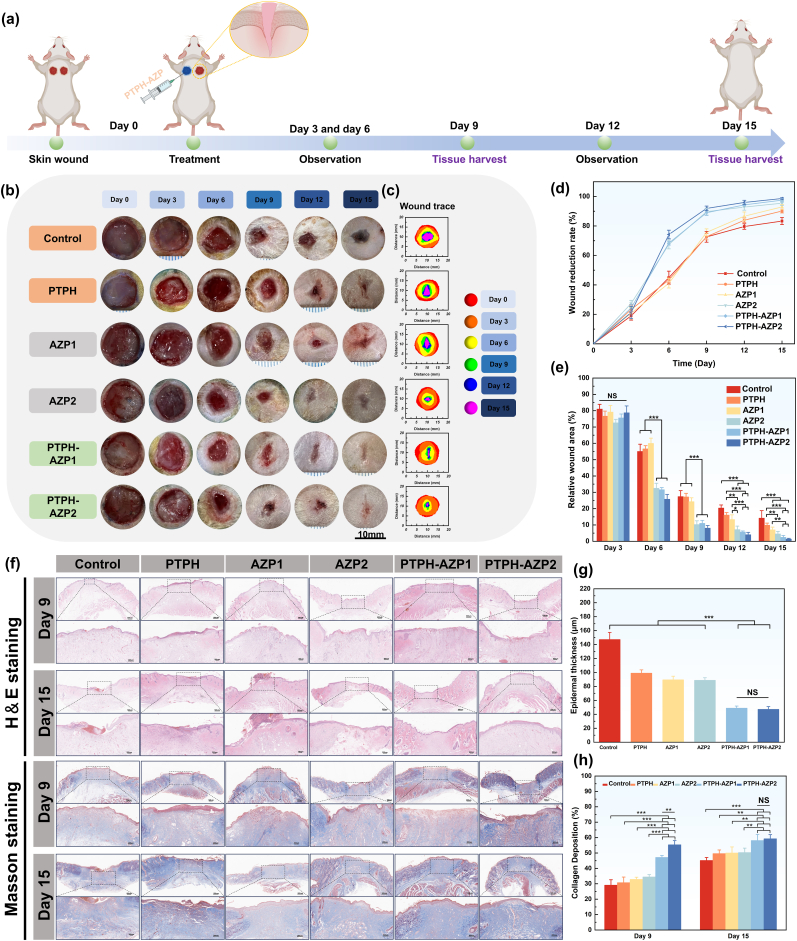
Assessment of hydrogel-promoted *in vivo* wound healing. (a) Experimental procedure for wound healing with different hydrogels. (b) Representative images of wounds at different times. (c) Traces of the wound healing process at different periods. (d) Quantification of wound contraction, and (e) quantification of the relative wound area. Histologic assessment of wounds from different groups. (f) H & E and Masson trichrome stained sections of wound tissues with different treatments on day 9 and day 15. (g) Epidermal thickness of each group at day 15. (h) Quantification of collagen deposition in each group at day 9 and day 15. Each value is presented as mean ± SD (n = 3). ∗*p* < 0.05, ∗∗*p* < 0.01, ∗∗∗*p* < 0.001.

The biocompatibility of biomaterials *in vivo* is critically important. To evaluate the *in vivo* biocompatibility of PTPH-AZP, we conducted subcutaneous implantation in rats, using saline as the control group. We assessed the inflammatory response at the implantation sites at both 3 and 7 days post-implantation (Fig. S9). As shown in Fig. S10, at both 3 and 7 days, the tissues surrounding the PTPH-AZP1 and PTPH-AZP2 implants exhibited no significant inflammatory response, with only a minimal infiltration of neutrophils, comparable to that of the saline control group. These results confirm that PTPH-AZP demonstrates excellent *in vivo* biocompatibility, supporting its potential application in further *in vivo* studies. *In vivo*, evaluation of the pro-vascularization and wound healing efficacy of PTPH-AZP hydrogels was conducted using a full-thickness skin defect model in SD rats (Fig. S11). The experimental treatment details are summarized in Fig. 6 (a). Full-thickness skin defects with a diameter of 10 mm were created, and different treatment groups were established: PTPH, AZP1 (AZP + PBS, 0.5 % w/w), AZP2 (AZP + PBS, 2 % w/w), PTPH-AZP1 (AZP, 0.5 % w/w), PTPH-AZP2 (AZP, 2 % w/w), and a Control group without treatment. Tegaderm™ film was applied to cover the wounds in all groups. Photographs of the wounds were taken on day 0, 3, 6, 9, 12, and 15, as shown in Fig. 6(b). To visualize the wound healing process, wound healing traces were drawn from the photographs (Fig. 6(c)), and changes in wound area were tracked to analyze wound healing efficiency (Fig. 6(d and e)). Quantitative analysis of wound area revealed no significant differences between the experimental groups on day 3. However, by day 6, the AZP2 group (healing rate of 67.48 ± 3.02 %), PTPH-AZP1 group (healing rate of 68.35 ± 1.29 %), and PTPH-AZP2 group (healing rate of 74.22 ± 2.92 %) exhibited significantly enhanced wound healing efficacy compared to the Control group (healing rate of 44.94 ± 4.41 %). This suggested superior wound healing promotion in the early stages of treatment by the high-concentration AZP (PBS) group and PTPH-AZP groups. By day 15, the PTPH-AZP1 group (97.53 ± 1.14 %) and PTPH-AZP2 group (98.64 ± 0.48 %) achieved significantly higher final healing rates compared to the Control group (83.32 ± 2.39 %). Interestingly, the AZP2 group (95.4 ± 1.53 %) exhibited comparable healing rates to the PTPH-AZP groups. This similar therapeutic effect in the AZP2 group could be attributed to the high concentration of AZP nanoclusters in the PBS solution, maintaining a Zn^2^⁺ concentration at the wound site that reached the threshold for promoting human umbilical vein endothelial cell (HUVEC) proliferation. From a clinical perspective, the challenge lies in maintaining the retention time and quantity of AZP-containing PBS at the wound site. In contrast, hydrogel groups exhibit a longer residence time at the wound site, enabling a more stable release of zinc ions. Therefore, PTPH-AZP groups are considered more suitable for use as wound dressings. These findings highlight the potential of PTPH-AZP to promote skin regeneration. Further investigations into the underlying mechanisms of wound healing were conducted through histological analysis, as described in the following section.

### 3.7 Histological evaluation of wound regeneration

Histological analysis of the regenerating epidermal tissue was performed utilizing H&E and Masson trichrome staining (Fig. 6 (f)). A marked reduction in the infiltration of neutrophils and lymphocytes at the wound periphery was observed in both the PTPH-AZP1 and PTPH-AZP2 treatment groups compared to the control group by day 9 post-wounding. At day 15, complete epidermal regeneration was observed in both PTPH-AZP treatment groups, while the control group exhibited persistent inflammatory cell infiltration. These findings suggest that PTPH-AZP effectively attenuates the inflammatory response and promotes the re-epithelialization process.

Furthermore, quantitative analysis revealed a significantly reduced epidermal thickness in the PTPH-AZP treated groups compared to the control group at day 15 (Fig. 6 (g)). The epidermal thickness in the PTPH-AZP groups closely resembled that of the adjacent unwounded skin. This observation is particularly significant given that excessive epidermal thickening is a hallmark of hypertrophic scar formation, further supporting the potential of PTPH-AZP in minimizing scar formation [58]. Notably, the presence of nascent glandular structures was observed in the PTPH-AZP1 group, indicative of functional skin regeneration. Masson trichrome staining revealed a substantial increase in collagen deposition at the wound site in both PTPH-AZP treatment groups compared to the control group at day 9 (Fig. 6 (h)). The PTPH-AZP2 group displayed the most pronounced collagen deposition, reaching 55.36 ± 2.70 %, representing a nearly 1.9 times increase compared to the control. This trend of enhanced collagen deposition in the PTPH-AZP treatment groups persisted through day 15, with the PTPH-AZP2 group exhibiting a collagen density of 59.26 ± 2.63 %, corresponding to a 1.31 times increase compared to the control. The accelerated re-epithelialization and robust collagen deposition observed in the PTPH-AZP treatment groups strongly suggest that PTPH-AZP holds considerable promise as a therapeutic agent for promoting wound healing by accelerating the deposition and maturation of the extracellular matrix.

Beyond collagen deposition, angiogenesis is another indispensable process during wound healing, as newly formed granulation tissue heavily relies on vascularization for nutrient supply [59]. CD31, a transmembrane protein actively expressed during early angiogenesis, serves as a reliable marker for assessing neovascularization. Its adhesive properties, mediating interactions between endothelial cells and between endothelial cells and leukocytes, are crucial for the formation and expansion of new blood vessels.

**Fig. 7:**
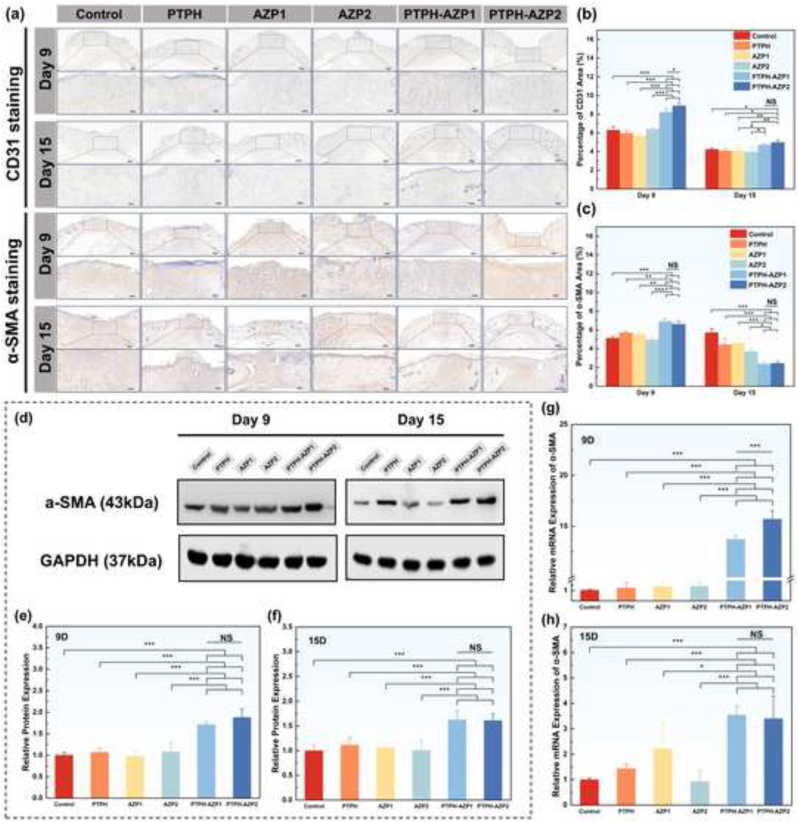
(a) IHC staining. Positive area analysis of (b) CD31 and (c)α-SMA. (d) Representative immunoblots of α-SMA protein in wound tissues on day 9 and 15, and their (e, f) semi-quantitative analysis. (g, h) qRT-PCR analysis of α-SMA mRNA expression levels in wound tissues on day 9 and 15. Each value is presented as mean ± SD (n = 3). ∗*p* < 0.05, ∗∗*p* < 0.01, ∗∗∗*p* < 0.001.

To further elucidate the underlying mechanisms of wound healing, we performed immunohistochemical analysis of CD31 expression (Fig. 7(a and b)). On day 9 post-wounding, the PTPH-AZP treatment groups exhibited significantly larger CD31-positive areas compared to other groups, particularly the PTPH-AZP2 group, which displayed a 1.41 times increase compared to the control group. This enhanced angiogenesis is attributed to the sustained release of Zn^2+^ from PTPH-AZP, which is known to promote neovascularization. By day 15, a decrease in CD31-positive areas was observed across all groups. Notably, while the PTPH-AZP groups displayed a reduction in the number of blood vessels, the remaining vessels exhibited a larger diameter compared to the control group. This observation can be explained by the maturation of CD31-positive capillaries into larger arterioles and venules during the later stages of wound healing, a crucial process for restoring normal skin tissue function. These findings suggest that PTPH-AZP accelerates wound healing by promoting rapid and extensive angiogenesis during the early-to-mid phase, effectively supplying the wound site with essential oxygen and nutrients to support the proliferation of newly formed granulation tissue. The subsequent reduction in capillary density in the later phase facilitates the restoration of normal skin function and potentially mitigates scar formation by preventing excessive vascularization. Angiogenesis and myofibroblast differentiation are recognized as critical processes for efficient wound healing and closure [60,61]. α-SMA, a key regulator of angiogenesis, serves as a prominent marker of myofibroblasts and is primarily localized within the stress fibers of these cells [62]. Consequently, α-SMA expression is strongly correlated with microvessel density and myofibroblast formation [63]. During the early stages of wound healing, robust α-SMA expression in myofibroblasts facilitates cytoskeletal reorganization and generates contractile forces, effectively drawing the wound edges together and accelerating closure. However, persistent overexpression of α-SMA in later stages can lead to excessive myofibroblast differentiation, potentially resulting in scar contracture [64,65]. As depicted in Fig. 7(a–c), the PTPH-AZP treatment groups displayed significantly larger α-SMA-positive areas compared to other groups on day 9 post-wounding. This observation indicates that PTPH-AZP effectively promotes wound contraction and the formation of mature blood vessels within the wound area during the early-to-mid phase of healing, thereby facilitating tissue regeneration. By day 15, a marked reduction in α-SMA-positive areas was observed in the PTPH-AZP treatment groups compared to other groups, suggesting the mitigation of excessive scar formation that could arise from prolonged α-SMA overexpression. In summary, these findings demonstrate a well-orchestrated healing process modulated by PTPH-AZP. Initially, PTPH-AZP promotes a surge in CD31-positive cells, driving rapid neovascularization to ensure adequate nutrient supply to the regenerating tissue. Concurrently, PTPH-AZP enhances α-SMA expression, facilitating myofibroblast differentiation, stress fiber formation, and ultimately, wound contraction. As the healing process progresses into the mid-to-late phase, neovascularization subsides as indicated by the decrease in CD31-positive vessels, and the wound tissue becomes more stabilized. To prevent excessive scarring, both CD31 and α-SMA expression are gradually downregulated, effectively preventing unnecessary vascularization and myofibroblast activity, ultimately promoting the restoration of normal skin function.

### 3.8 Western blot and qRT-PCR analysis

To delve into the molecular mechanisms underlying the wound healing properties of PTPH-AZP, we investigated its influence on the expression of α-SMA, a key player in wound healing processes. α-SMA, a hallmark of myofibroblast differentiation, plays a pivotal role in wound contraction and extracellular matrix (ECM) deposition. Western blot analysis was employed to visualize and semi-quantitatively assess α-SMA protein expression levels in skin samples from each group (Fig. 7(d–f)). Our findings revealed a significant upregulation of α-SMA protein in the PTPH-AZP treatment groups compared to the control group on day 9 post-wounding. Specifically, PTPH-AZP1 and PTPH-AZP2 groups exhibited approximately 1.71 times and 1.88 times increases in α-SMA protein expression relative to the control, respectively. While a decline in expression was observed by day 15, the PTPH-AZP treatment groups still maintained elevated levels of α-SMA protein at 1.62 times and 1.60 times compared to the control. To further corroborate the impact of PTPH-AZP on α-SMA expression, we performed qRT-PCR to quantify α-SMA mRNA levels at different stages of wound healing. Consistent with the protein data, on day 9, PTPH-AZP1 and PTPH-AZP2 groups displayed significantly higher α-SMA mRNA expression levels, reaching 13.74 times and 15.69 times that of the control group, respectively. These levels subsequently decreased to 3.54 times and 3.40 times by day 15 (Fig. 7(g–h)). These results collectively suggest that PTPH-AZP accelerates wound healing, at least in part, through the upregulation of α-SMA. This heightened α-SMA expression likely promotes angiogenesis and wound contraction during the early stages of wound healing. The subsequent downregulation of α-SMA expression observed in later stages may contribute to the prevention of excessive scar formation.

## 4 Conclusion

In this study, we developed an injectable thermosensitive hydrogel, PTPH-AZP, based on aliphatic polycarbonate and loaded with AZP. *In vitro* experiments demonstrated that PTPH-AZP exhibits excellent biocompatibility and releases Zn^2+^ at concentrations conducive to effectively promoting the proliferation, migration, and tube formation of HUVECs. *In vivo* experimental results indicated that PTPH-AZP enhances wound healing by upregulating the expression of CD31 and α-SMA to promote angiogenesis, modulating myofibroblast activity for effective wound closure, and reducing excessive scar tissue formation, ultimately accelerating the wound closure process. Given its outstanding biocompatibility and ability to promote skin tissue regeneration, PTPH-AZP shows substantial clinical application potential. Future research will focus on elucidating the precise molecular mechanisms underlying these beneficial effects and evaluating the efficacy and safety of PTPH-AZP in preclinical models.

## CRediT authorship contribution statement

**Siwen Chen:** Writing – original draft, Formal analysis, Data curation, Conceptualization. **Yutong Li:** Writing – original draft, Software. **Sihang Ren:** Methodology, Data curation. **Yuanyuan Yang:** Methodology. **Zhipeng Hou:** Supervision, Methodology. **Siyu Han:** Formal analysis. **Wanhong Zhang:** Software. **Jing Guo:** Supervision. **Jianshe Hu:** Writing – review & editing, Supervision, Funding acquisition, Conceptualization. **Xing Zhang:** Writing – review & editing, Funding acquisition, Conceptualization. **Liqun Yang:** Writing – review & editing, Funding acquisition, Conceptualization.

## Declaration of competing interest

The authors declare that they have no known competing financial interests or personal relationships that could have appeared to influence the work reported in this paper.

## Data Availability

Data will be made available on request.
