# Supplementary material for: Amorphous zinc phosphate nanoclusters loaded polycarbonate thermosensitive hydrogel: An innovative strategy for promoting wound healing
Source: Mater Today Bio. 2024 Sep 24;29:101266. doi: 10.1016/j.mtbio.2024.101266 (PMC11460505; doi:10.1016/j.mtbio.2024.101266)
Supplement: Multimedia component 1 [file mmc1.docx]

**Amorphous Zinc Phosphate Nanoclusters Loaded Polycarbonate Thermosensitive Hydrogel: An Innovative Strategy for Promoting Wound Healing**

Siwen Chen^1, 2^, Yutong Li^3^, Sihang Ren^4^, Yuanyuan Yang^2^, Zhipeng Hou^2^, Siyu Han^1^, Wanhong Zhang^1^, Jing Guo^5^, Jianshe Hu^1,^ *, Xing Zhang^3,^ *, Liqun Yang^2,^ *

^1^ Center for Molecular Science and Engineering, College of Science, Northeastern University, Shenyang 110819, P.R. China

^2^ Research Center for Biomedical Materials, Engineering Research Center of Ministry of Education for Minimally Invasive Gastrointestinal Endoscopic Techniques, Shengjing Hospital of China Medical University, Shenyang, 110004, P.R. China

^3^ Institute of Metal Research, Chinese Academy of Sciences, Shenyang 110016, P.R. China

^4^ Department of Plastic Surgery, The Second Hospital of Dalian Medical University, Dalian 116027, P.R.China

^5^ Liaoning Research Institute for Eugenic Birth & Fertility, China Medical University, Shenyang 110031, P.R. China

*****Corresponding Author:

Liqun Yang, E-mail: yangliqun@sj-hospital.org

Xing Zhang, E-mail: [xingzhang@imr.ac.cn](mailto:xingzhang@imr.ac.cn)

Jianshe Hu, E-mail: [hujs@mail.neu.edu.cn](mailto:hujs@mail.neu.edu.cn)

**S1 In vitro degradation experiment of PTPH**

A volume of 0.5 mL of PTPH was placed in a 5 mL centrifuge tube and incubated at 37°C for 10 minutes to facilitate hydrogel formation. The hydrogel was then divided into two groups: one group was immersed in 1.5 mL of PBS, while the other was immersed in 1.5 mL of a 0.1% (w/w) lipase solution. The solutions were refreshed every three days. The degradation experiment was conducted in a thermostatic shaker set at 37°C with a shaking speed of 60 rpm, gently agitating the samples for 8 hours per day. At predetermined time points (0.5, 1, 3, 5, and 7 days), triplicate samples from each group were collected, dried under vacuum to a constant weight, and the weight loss was determined.

$$Mass loss \left( \% \right)=\frac{W_{0}-W_{1}}{W_{0}}\times100$$

$W_{0}$ is the initial weight of the polymer in the sample before degradation, and $W_{1}$ is the dry weight of the sample after degradation.

**S2 Cell culture, proliferation, scratching assay, and tube formation**

HUVECs and mouse fibroblasts (L929 cells) from the laboratory were cultured in Dulbecco's Modified Eagle's Medium (DMEM high glucose, Sigma, USA) supplemented with 10% fetal bovine serum (FBS, VivaCell, Shanghai, China) and 1% penicillin-streptomycin (Gibco, USA). The cells were maintained at 37°C in an incubator with 5% CO_2_.

HUVEC and L929 cells proliferation was evaluated using the Cell Counting Kit-8 (CCK-8, Biosharp, China). After sterilization, the PTPH-AZP with varying AZP ratios were co-cultured with the cell culture medium for 24 h to obtain the impregnation solution of the PTPH-AZP culture medium. HUVECs and L929 cells were seeded in a 96-well plate at a density of 1×10^4^ cells per well and co-cultured with the impregnation solution medium of PTPH-AZP for 72 h. Subsequently, the culture medium in each well was replaced with a 10% CCK-8 solution and incubated for an additional 2 h. Optical density (OD) values were measured using a microplate reader (Multiskan GO, Thermo Fisher Scientific, USA) to quantify cell viability.

The steps of the cell scratching experiment are as follows: HUVECs were cultured in a 12-well plate at a density of 1×10^5^ cells per well. After 24 h of culture, a 200 μL pipette tip was used to create a scratch on the surface of the cell layer to simulate a wound. Different proportions of AZP in the PTPH-AZP culture medium impregnation solution were then added and co-incubated with the HUVECs for 12 h. Subsequently, the culture medium was aspirated, followed by three PBS washes, and cells were fixed with 4% paraformaldehyde for 30 min. The healing of the HUVECs scratch was observed and photographed using an inverted optical microscope（Nikon Ti Eclipse）, and the wound healing rate was calculated using the following equation:

$$Wound healing rate =\frac{\left( A_{0}-A_{1} \right)}{A_{0}}\times100\%$$

Where A_0_ is the initial wound area, and A_1_ is the wound area after 24 h of co-culture.

The steps of the tube formation experiment are as follows: following standard procedures, Cultrex BME (R&D Systems, USA) was thawed and added to a 24-well plate (80 μL) and gelled at 37°C for 2 h. Subsequently, different proportions of AZP in PTPH-AZP impregnation solution medium (200 μL) were transferred into each well, followed by the addition of HUVECs (1×105 cells/well). In the control group, only a blank cell culture medium was added. Tube formation was periodically observed, and images were captured at 6 h using an inverted optical microscope (Nikon Ti Eclipse). Images of at least five different areas were collected for each group. The number of tubes, junctions, nodes, and the total tube length were analyzed using Image J software.

**S3 Hemolysis tests**

In brief, fresh rat blood was centrifuged at 1500 rpm for 5 min to separate the red blood cells. The red blood cells were then washed multiple times with saline until the supernatant was clear. At the same time, PTP-3 and AZP were dispersed in normal saline (PTP-3 concentration: 100 mg/mL, AZP concentration: 10 mg/mL). A mixture was prepared by adding 0.1 mL of red blood cells to 0.8 mL of normal saline, followed by the addition of 50 μL of PTP-AZP solution. The mixture was incubated at 37°C for 1 h. H_2_O was used as a positive control, while normal saline served as a negative control. After incubation, the mixture was centrifuged for 5 min, and 100 μL of the supernatant was transferred into a 96-well microplate. The absorbance of the solution at 545 nm was measured using a microplate reader. Each group had at least three parallel samples. The hemolysis rate was calculated using the following equation:

$$Hemolysis rate =({A_{s}-A_{n})}/{(A_{p}-A_{n})}\times100\%$$

Where A_s_, A_p_ and A_n_ are the absorbance of the hydrogel experimental group, positive control group, and negative control group, respectively. Three parallel samples were set up for each group.

**S4 Efficacy evaluation of PTPH-AZP in treating skin defects of SD rats**

36 male SD rats weighing 280-320 g (Liaoning Changsheng Biotechnology Co., Ltd.) were selected as experimental animals and randomly divided into six groups, each comprising six rats. Following standard anesthesia procedures, the back hair of rats was removed, and a full-thickness skin defect area with a diameter of 10 mm was created on each side of their backs using surgical scissors. Silicone pads (Ø15 × 1 mm) were fixed on the wounds after surgery to prevent contraction. The six groups were named as follows: Control group, PTPH group, AZP1 group (AZP+PBS, 0.5%, w/w), AZP2 group (AZP+PBS, 2%, w/w), PTPH-AZP1 (AZP, 0.5%, w/w) group, and PTPH-AZP2 (AZP, 2%, w/w) group. PTPH, AZP1, AZP2, PTPH-AZP1, and PTPH-AZP2 were injected into the wounds to form wound dressings, which were subsequently covered with Tegaderm™ film. The wounds of the control group were directly covered with Tegaderm™ film without any further treatment. On day 3, 6, 9, 12, and 15, the wound areas of all animals were assessed and documented using a digital camera. The wound areas for each group were measured and analyzed using Image J software. The wound healing rate was calculated using the following equation:

$$Wound healing=\frac{(S_{0}-S_{n})}{S_{0}}\times100\%$$

Where S_0_ and S_n_ represent the initial wound area and the wound area at different time points, respectively.


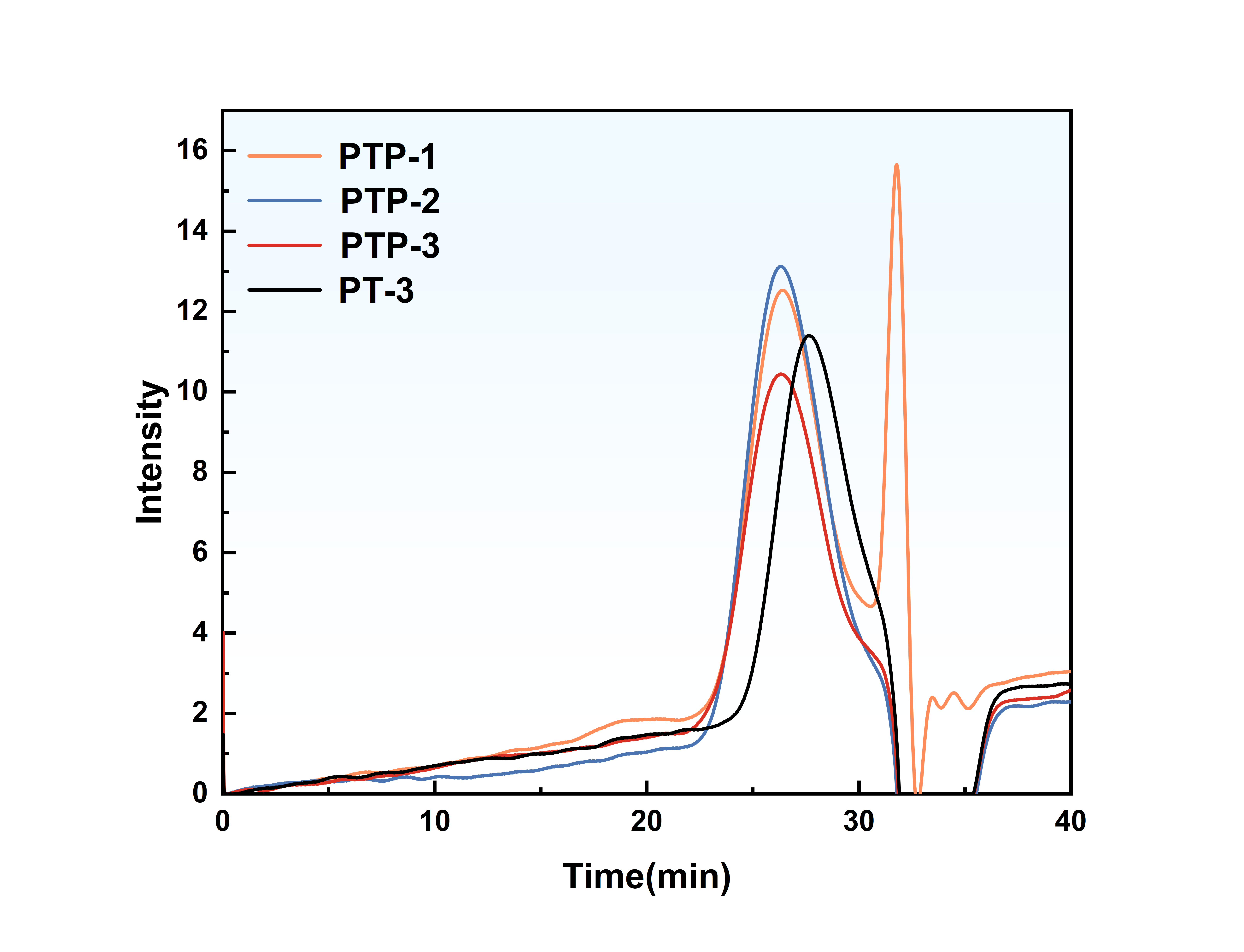


Fig.S1


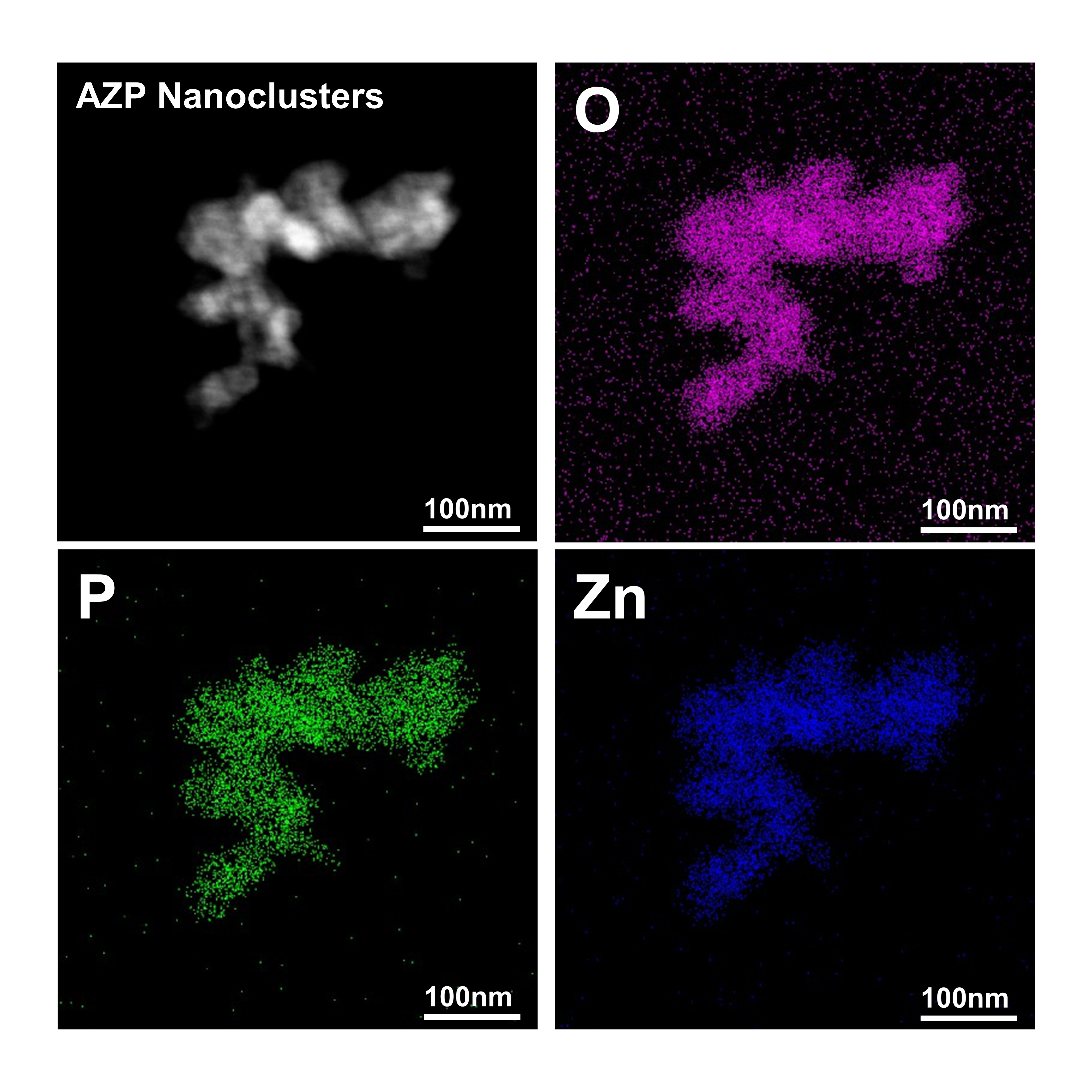


Fig.S2


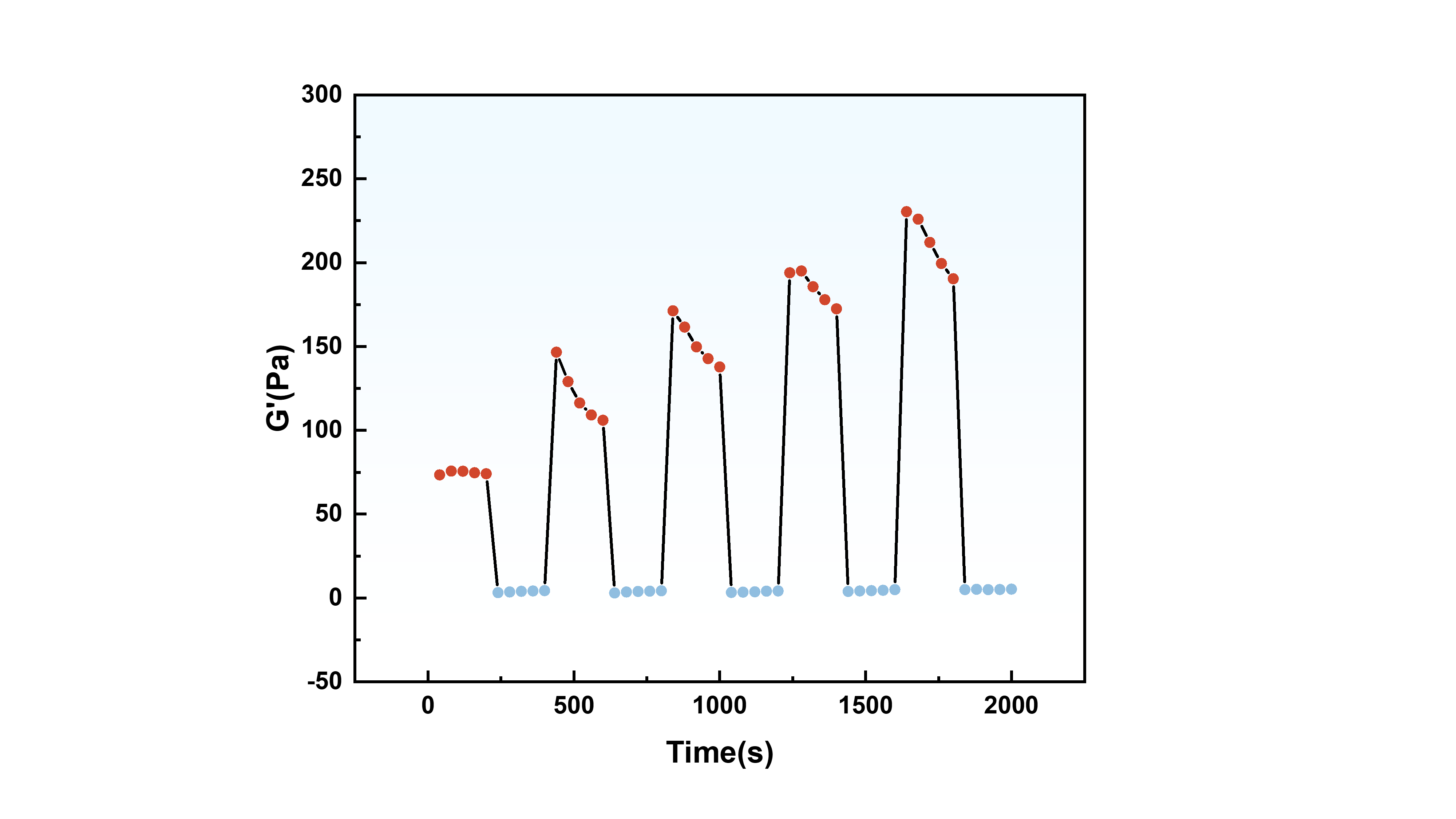


Fig.S3


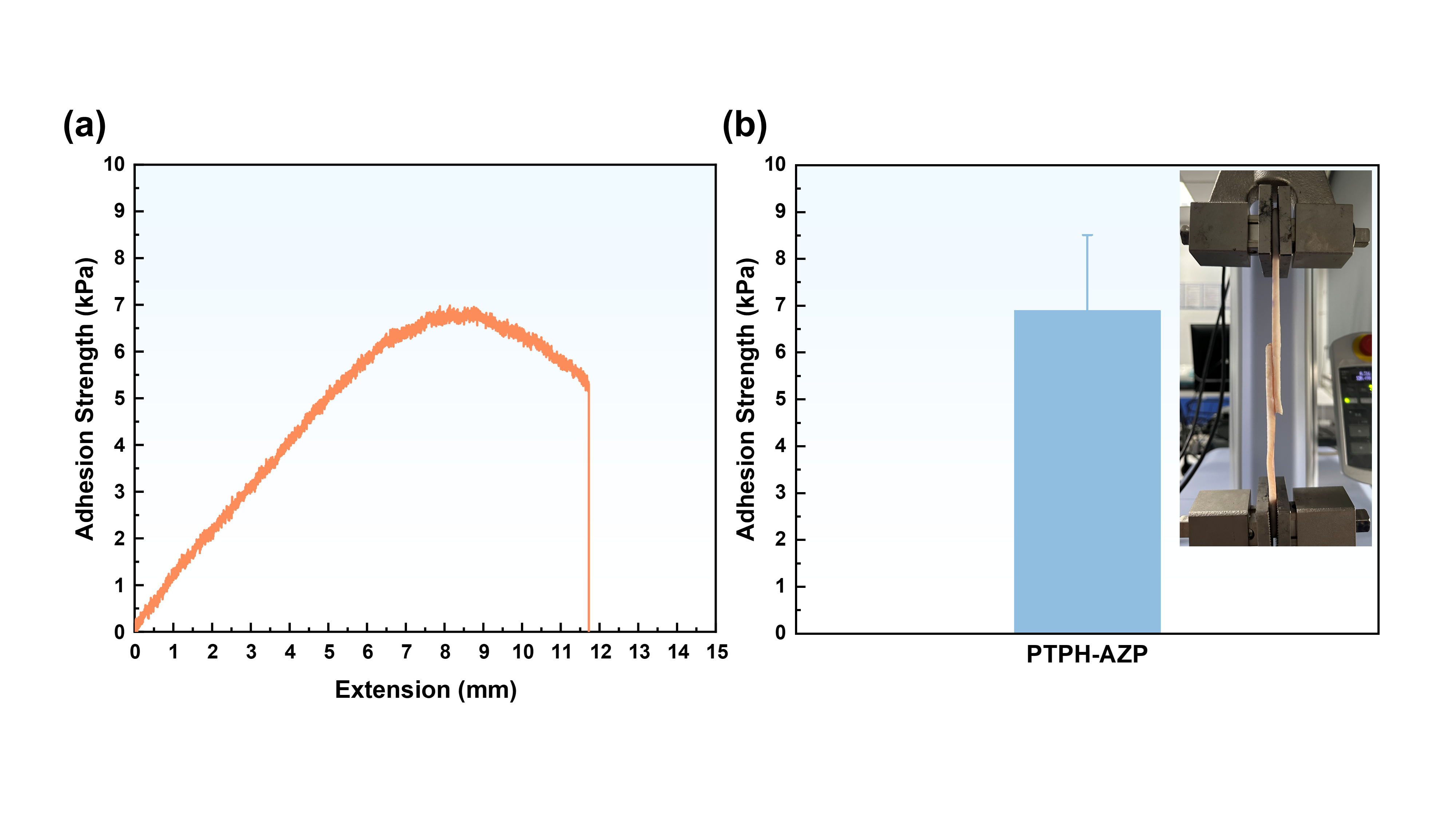


Fig.S4


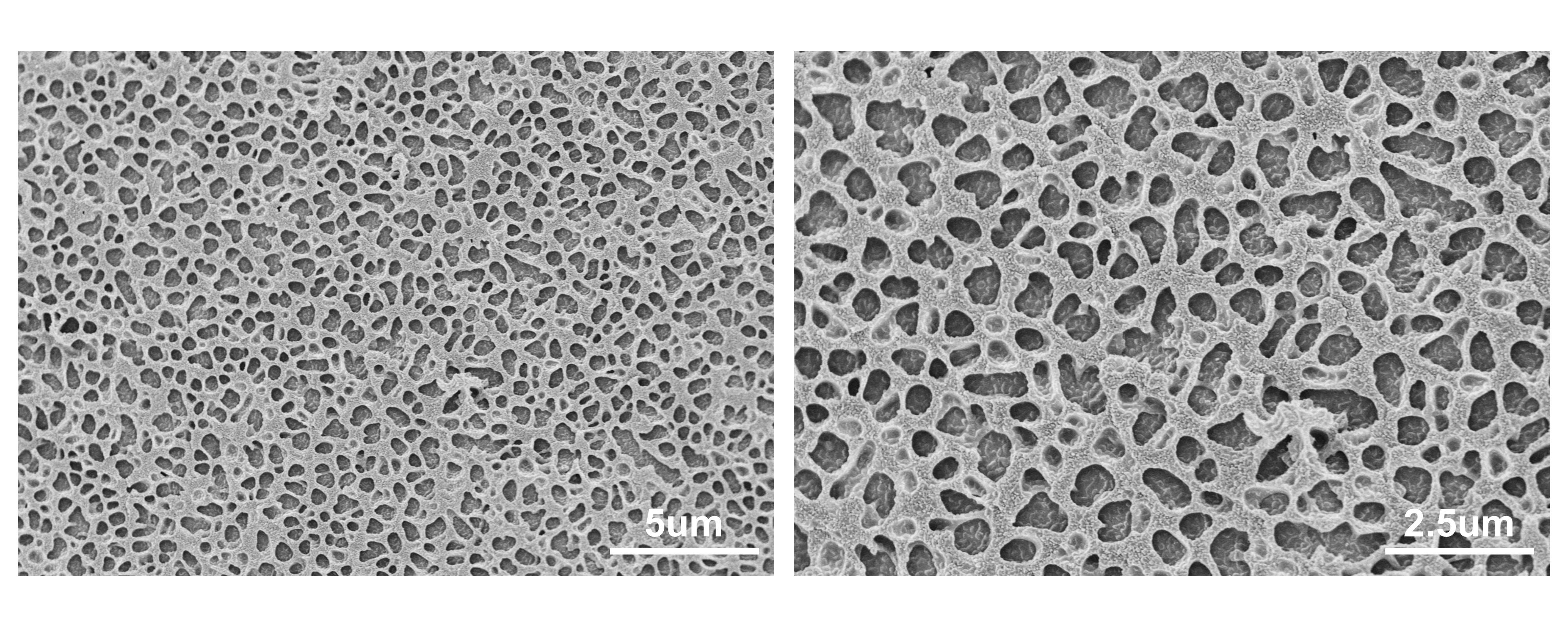


Fig.S5


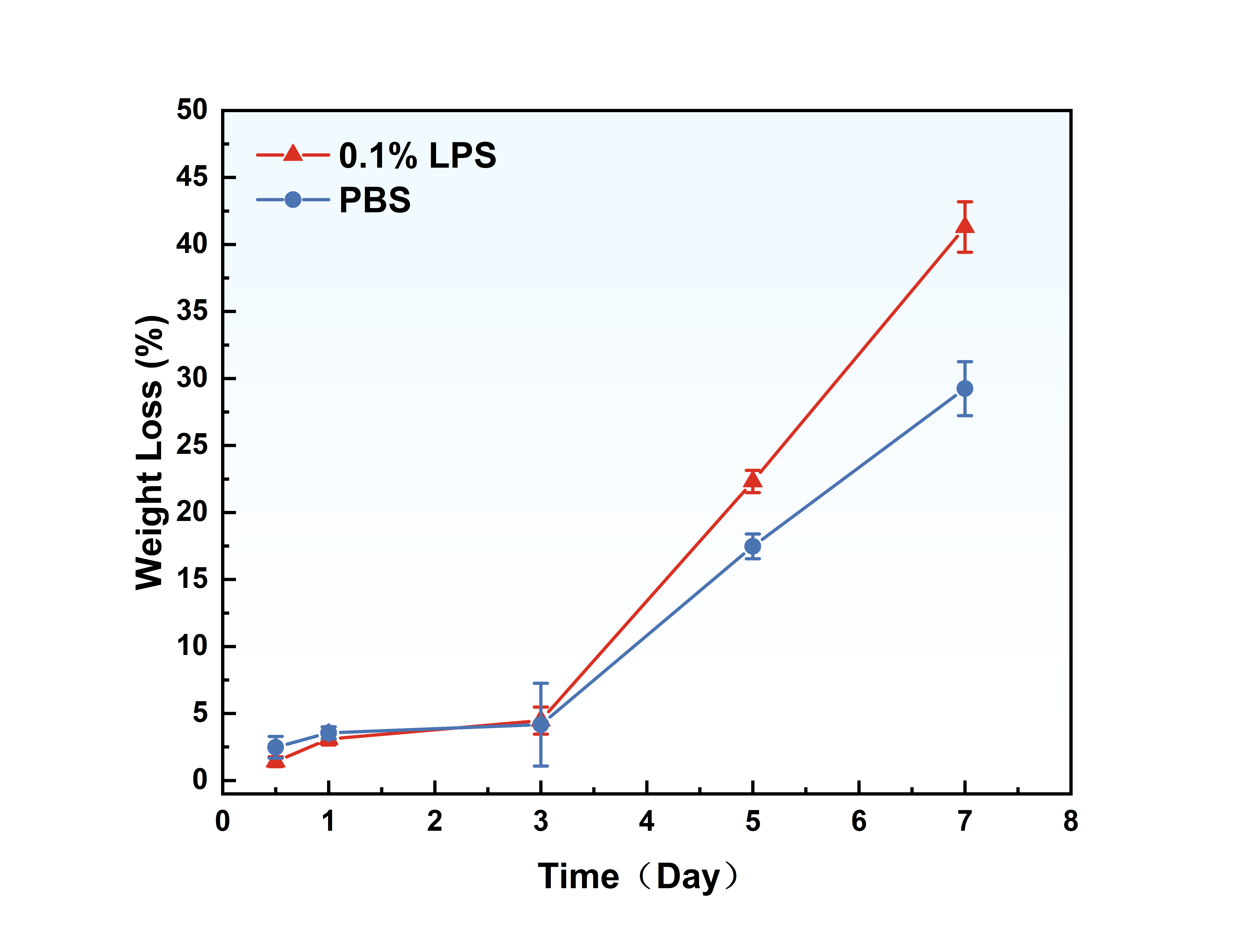


Fig.S6


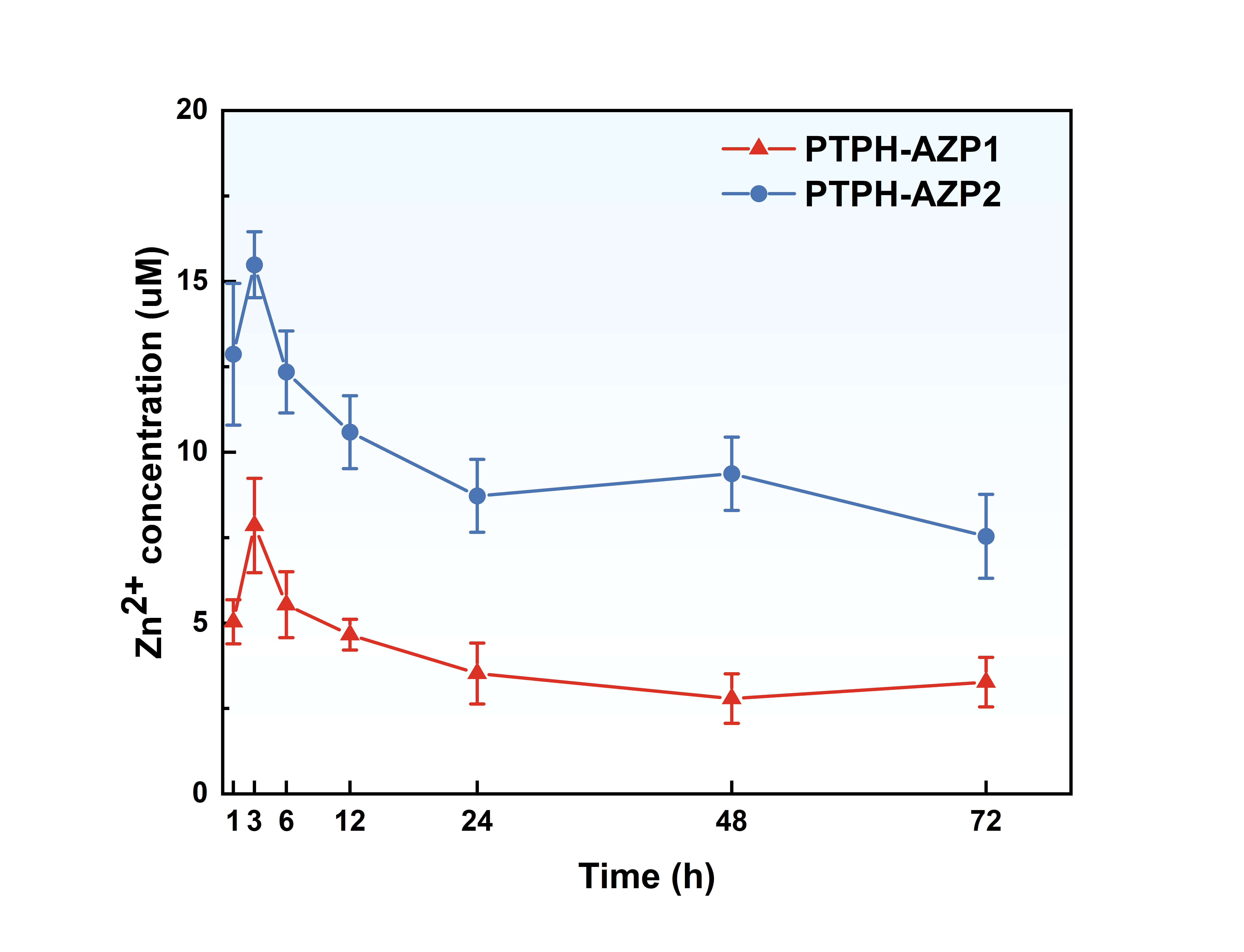


Fig.S7


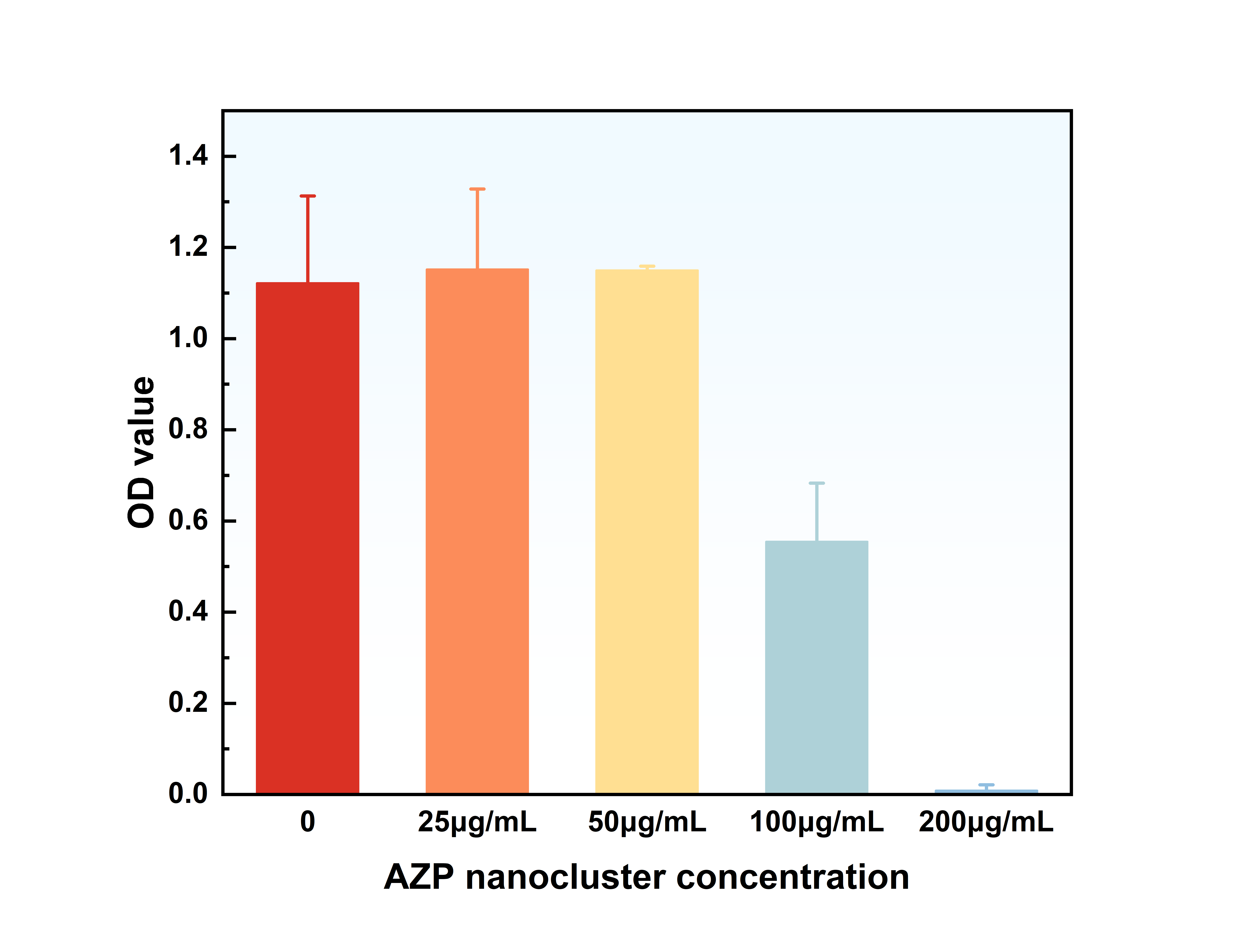


Fig.S8


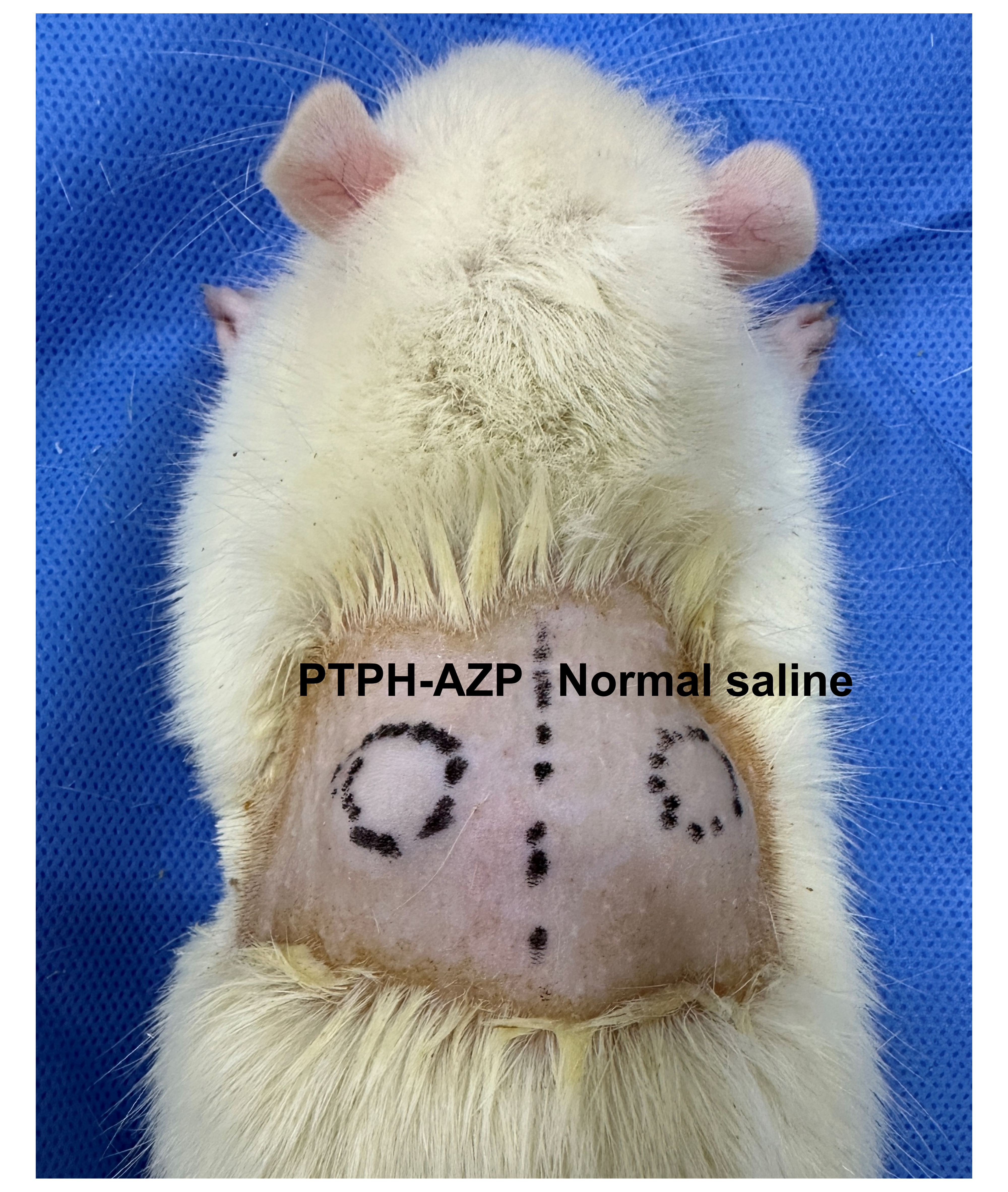


Fig.S9


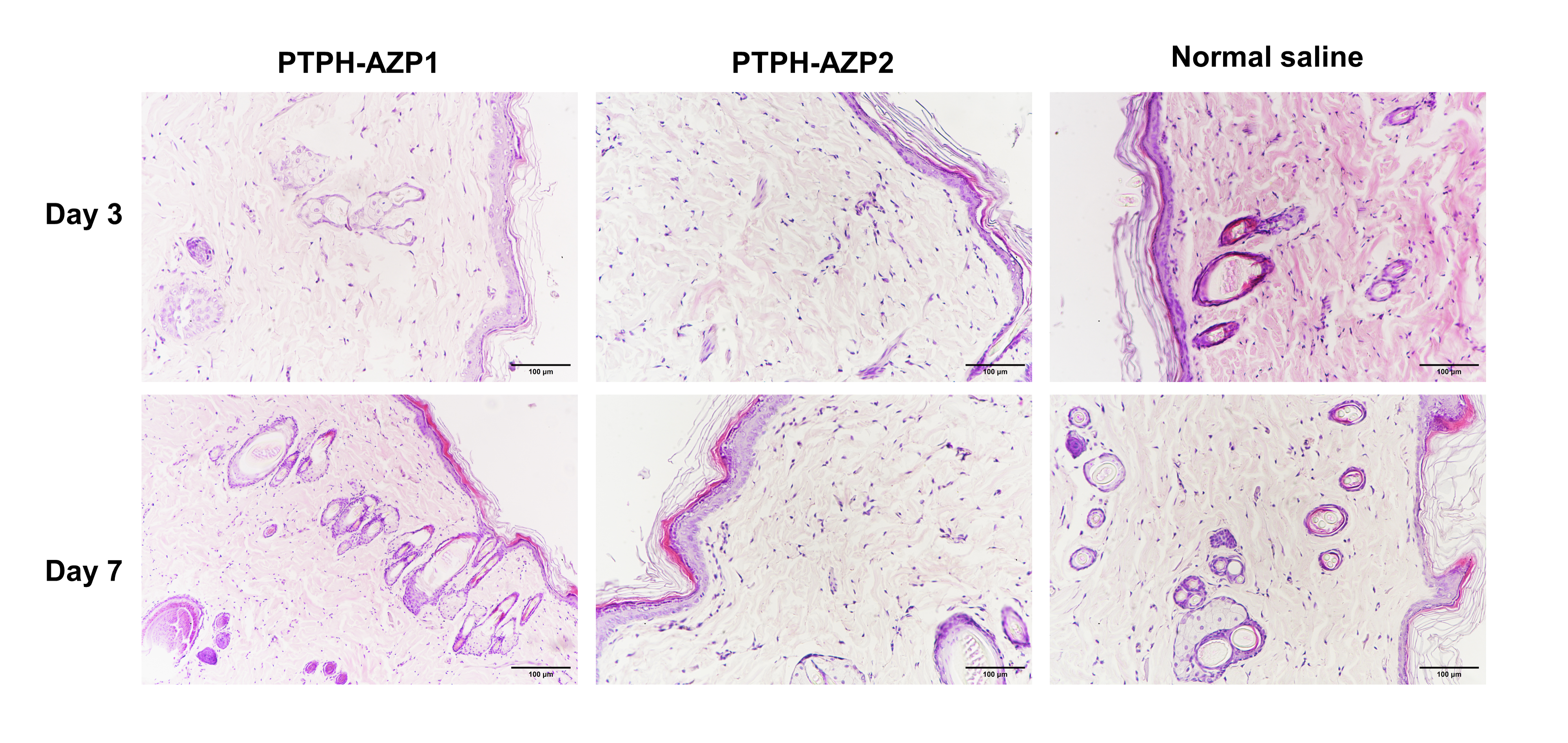


Fig.S10


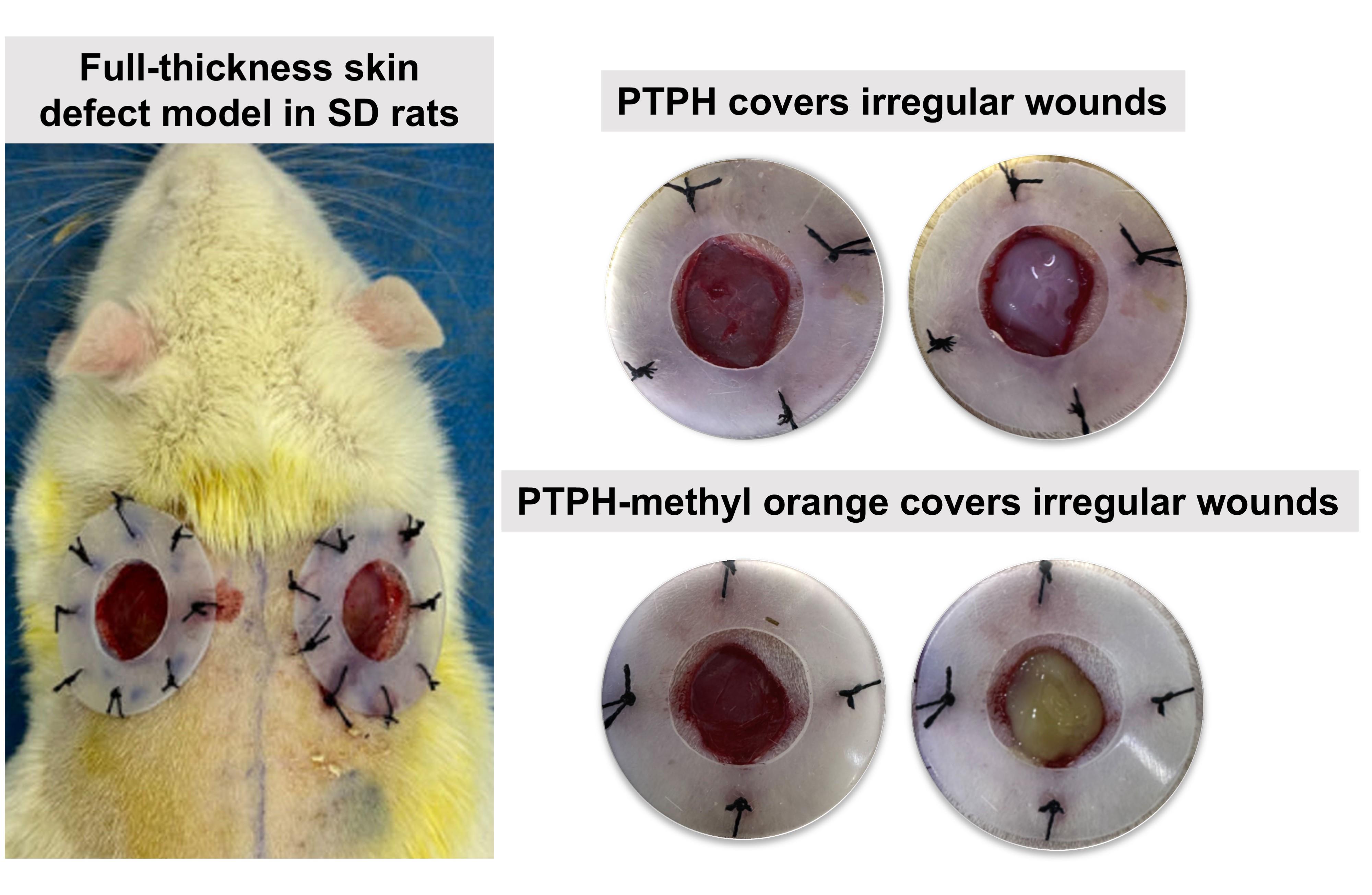


Fig.S11
